# Matrix M H5N1 Vaccine Induces Cross-H5 Clade Humoral Immune Responses in a Randomized Clinical Trial and Provides Protection from Highly Pathogenic Influenza Challenge in Ferrets

**DOI:** 10.1371/journal.pone.0131652

**Published:** 2015-07-06

**Authors:** Rebecca J. Cox, Diane Major, Gabriel Pedersen, Rishi D. Pathirana, Katja Hoschler, Kate Guilfoyle, Sarah Roseby, Geir Bredholt, Jörg Assmus, Lucy Breakwell, Laura Campitelli, Haakon Sjursen

**Affiliations:** 1 Influenza Centre, Department of Clinical Science, University of Bergen, Bergen, Norway; 2 Department of Research and Development, Haukeland University Hospital, Bergen, Norway; 3 Jebsen Centre for Influenza Vaccine Research, University of Bergen, Bergen, Norway; 4 National Institute for Biological Standards and Control (NIBSC), Potters Bar, United Kingdom; 5 Respiratory Unit, Public Health England (PHE) Colindale, London, United Kingdom; 6 Istituto Superiore di Sanita (ISS), Rome, Italy; 7 Section for Infectious Diseases, Medical Department, Haukeland University Hospital, Bergen, Norway; Glaxo Smith Kline, DENMARK

## Abstract

**Background and Methods:**

Highly pathogenic avian influenza (HPAI) viruses constitute a pandemic threat and the development of effective vaccines is a global priority. Sixty adults were recruited into a randomized clinical trial and were intramuscularly immunized with two virosomal vaccine H5N1 (NIBRG-14) doses (21 days apart) of 30μg HA alone or 1.5, 7.5 or 30μg HA adjuvanted with Matrix M. The kinetics and longevity of the serological responses against NIBRG-14 were determined by haemagglutination inhibition (HI), single radial haemolysis (SRH), microneutralization (MN) and ELISA assays. The cross-H5 clade responses in sera were determined by HI and the antibody-secreting (ASC) cell ELISPOT assays. The protective efficacy of the vaccine against homologous HPAI challenge was evaluated in ferrets.

**Results:**

The serological responses against the homologous and cross-reactive strains generally peaked one week after the second dose, and formulation with Matrix M augmented the responses. The NIBRG-14-specific seroprotection rates fell significantly by six months and were low against cross-reactive strains although the adjuvant appeared to prolong the longevity of the protective responses in some subjects. By 12 months post-vaccination, nearly all vaccinees had NIBRG-14-specific antibody titres below the protective thresholds. The Matrix M adjuvant was shown to greatly improve ASC and serum IgG responses following vaccination. In a HPAI ferret challenge model, the vaccine protected the animals from febrile responses, severe weight loss and local and systemic spread of the virus.

**Conclusion:**

Our findings show that the Matrix M-adjuvanted virosomal H5N1 vaccine is a promising pre-pandemic vaccine candidate.

**Trial Registration:**

ClinicalTrials.gov NCT00868218

## Introduction

HPAI virus subtype H5N1 continues to be a serious threat to animal and human health [1]. Human cases of H5N1 are rare, however infection results in high fatality rates. Between 2003 and January 2015, the World Health Organization (WHO) has reported 694 cases of human H5N1 infections in 16 countries with 402 deaths [2]. H5N1 infections have mainly been detected in the Asian continent, with Indonesia (197 cases), Egypt (203 cases), Viet Nam (127 cases), Cambodia (56 cases), China (47 cases) and Thailand (25 cases) reporting the highest number of cases [2]. The first known fatal case of human H5N1 in North America was imported to Canada from Asia in 2014, highlighting the potential for trans-continental spread of these viruses.

Vaccination is the main intervention to reduce morbidity and mortality associated with influenza H5N1 infections. The Influenza H5N1 virus is a poor immunogen compared with seasonal strains or pandemic 2009 H1N1 and humans do not have pre-existing immunity to H5 viruses. Human clinical trials have shown that at least two doses of H5N1 vaccines formulated with effective adjuvants are needed to elicit protective immune responses [3]. Very few adjuvants are currently used in human vaccines with aluminium salts being the most widely used, but these do not augment the response to H5N1. Novel adjuvants such as proprietary oil-in-water emulsions (AS03, MF59) have shown great potential for antigen dose-sparing and augmenting immune response to homologous and cross-reactive strains after H5 vaccination (reviewed by [3]). Immune stimulating complex (ISCOM) technology is another promising adjuvant with a good safety profile in humans and has been shown to augment the antibody and cellular immune response after vaccination (summarized in [4]). ISCOMs are formed by strong cholesterol binding to *Quillaja* saponins (*Quillaja saponaria* Molina) forming 12nm rings, which in turn are held together by lipid based hydrophobic interactions to form spherical particles 40nm in diameter. Matrix M is a third generation ISCOM and is a mix of Matrix C, which is a highly active but slightly reactogenic sapononin, and Matrix A, which is a weaker but well tolerated saponin [4]. In a phase I clinical trial involving 60 healthy adults, we have demonstrated that a Matrix M-adjuvanted H5N1 (NIBRG-14) virosomal vaccine has an acceptable safety profile, capacity for antigen dose sparing and induce a balanced Th1/Th2 antibody and cellular responses, including multifunctional T cells [5–7]. Furthermore, this vaccine elicited protection against HPAI H5N1 virus challenge in murine pre-clinical studies [6, 8]. In the current study, we report in detail the early kinetics of the homologous and cross-H5 clade immune response and the long-term effectiveness of the NIBRG-14 H5N1 influenza vaccine formulated with or without the Matrix M adjuvant in humans. Furthermore, we tested the protective efficacy of the NIBRG-14 H5N1 vaccine against challenge with highly pathogenic avian influenza virus infection in a ferret infection model, which is a well-established model for influenza research (reviewed by [9]).

## Materials and Methods

### Study participants and ethics statement

A phase I open label clinical trial was conducted in 60 healthy adult subjects (20–49 years old) at the Haukeland University Hospital, Bergen, Norway. Prior to being enrolled in the study, all participants provided written informed consent and were screened for normal haematology, biochemistry and immunology parameters. The full study demographics and exclusion criteria were reported earlier [5]. The study was approved by the local regional ethics committee of Northern Norway (Regional komité for medisinsk og helsefaglig forskningsetikk, Nord-Norge, REK Nord) and the Norwegian Medicines Agency and registered in the European Clinical Trials Database (EudraCT no. 2008-006940-20), the WHO Initiative for Vaccine Research [10] and National Institute for Health (NIH, Clinical trials.gov, Study: NCT00868218).

### Vaccine and Matrix M adjuvant

The virus strain used for the vaccine is the reverse genetics NIBRG-14 strain, which is a reassortant between the human A/Vietnam/1194/2004 (H5N1) and A/Puerto Rico/8/34 (H1N1) (PR8) strains. The influenza virosomal vaccine was produced under Good Manufacturing Practice by Crucell Berna Biotech (the Netherlands) as described previously [5]. The vaccine (1.5, 7.5, or 30μg HA) was formulated with Matrix-M (50μg, Novavax, MD, USA) in phosphate buffered saline (PBS) and filled into single use syringes (0.5ml per syringe) and stored at 4°C until used.

### Study design

We recruited 60 healthy adult volunteers (mean age 31 years old, 60% female, between 24.03.2009 and 23.06.2009) and the clinical investigator randomly allocated subjects into four groups of 15 by drawing lots. The random allocation was concealed from all except the clinical investigator until completion of the immunological assays. A dose escalation study was performed where five subjects from each group were vaccinated twice, 21 days (±1) apart with the virosomal H5N1 vaccine alone (30μg HA) or with vaccine (1.5, 7.5 or 30μg HA) formulated with Matrix-M (50μg) adjuvant. This was followed by immunization of the remaining 10 subjects from each group using the same vaccination schedule. A sample size of 15 was determined by the clinical investigator as the minimum number needed to detect statistically significant differences between the vaccine groups. A group size of 15 is common for first in humans, phase I trials, however, a larger phase II/III trial would be required with higher statistical power to provide further safety and immunogenicity data. The inclusion and exclusion criteria were reviewed for each subject before administration of each dose of vaccine. Blood samples were collected at 7, 14 and 21 days after each dose of vaccine and 180 and 365 days after the first vaccination. All blood (serum) samples were aliquoted and stored at -80°C until used in serological assays as described previously [5]. Peripheral blood mononuclear cells (PBMCs) were harvested from Cell Separation Tubes (CPT, BD Biosciences) according to manufacturer’s instructions and used immediately in the ELISPOT assays.

### Serological assays

All serum samples collected pre- and post-vaccination were tested in the haemagglutination inhibition (HI) assay using the egg grown viruses A/Vietnam/1194/2004 (NIBRG-14, clade 1), A/Indonesia/05/2005 (IBCDC-RG2, clade 2, subclade 1.3.2), A/turkey/Turkey/1/2005 (NIBRG-23, clade 2, subclade 2.2.1) and A/Cambodia/R0405050/2007 (NIBRG-88, clade 1, subclade 1) as described previously [5]. The microneutralization (MN) and single radial hemolysis (SRH) assays were performed against the homologous NIBRG-14 strain by Health Protection England, United Kingdom and Istituto Superiore di Sanita, Italy, respectively as described before [11, 12]. All serum samples were run at least in duplicate in the serological assays.

### The antibody secreting cell (ASC) response

The number of ASCs were evaluated by Enzyme Linked ImmunoSPOT assay (ELISPOT) as described previously [13] with some modifications. Briefly, 96 well ELISPOT plates (Multiscreen HA, Millipore) were coated with 2μg/ml A/Vietnam/1194/2004 (NIBRG-14, clade 1), A/turkey/Turkey/1/2005 (NIBRG-23, clade 2, subclade 2.2.1), A/Cambodia/R0405050/2007 (NIBRG-88, clade 1, subclade 1) or A/Anhui/1/05 (RG6, clade 2, subclade 3.4) whole virus overnight at 4°C. PBMCs (5×10^5^ cells) were added to duplicate wells and incubated overnight at 37°C and 5% CO2. Antibodies (IgG, IgA or IgM) secreted by the PBMCs were detected by incubation with goat anti-human class-specific antibodies for 2h at room temperature. After development with the substrate (9-amino 3-ethyl carbazole), the number of spots was calculated using an ELISPOT Immunoscan reader and the ImmunoSpot software (CTL-Europe GmbH, Germany).

### Memory B cell response

The NIBRG-14-specific IgG memory B cell (B_mem_) response after vaccination was quantified by ELISPOT as described elsewhere [14]. The ELISPOT assay was conducted as described above with NIBRG-14 virosomes coated on the ELISPOT plates. Results are presented as virus-specific IgG memory B cells per 1 × 10^6^ PBMCs.

### The serum IgG response

The homologous influenza-specific IgG antibodies were quantified by Enzyme Linked Immonosorbant assay (ELISA) as previously described [15]. Briefly, ELISA plates were coated with 2μg/ml influenza virosomal vaccine and capture goat anti-human IgG antibodies (1mg/ml) overnight at 4°C. Serially diluted human sera and immunoglobulin standards were incubated for 2h at room temperature followed by one hour incubation with biotin-conjugated goat anti-human IgG (1/1000 dilution) and one hour incubation with extravidin peroxidase (1/1000). The antibody concentrations were calculated using the IgG standards and linear regression of the log-transformed readings.

### Vaccination and viral challenge of ferrets

Adult male ferrets (Mustela putorius furo) at 7–9 months of age were randomly assigned to one of five study groups (10 ferrets per group). A sample size of 10 was determined by the chief investigator as the minimum number needed to detect statistically significant differences between the vaccine groups. Ferrets were housed in open pens after vaccination and then transferred to individual cages prior to challenge. Animals were fed twice daily with ferret breeding and maintenance diet. All ferrets were seronegative as determined by HI assays (HI titres <10) to currently circulating influenza H3, H1 and B strains. The study was approved by National Institute for Biological Standards and Control (NIBSC) Animal Welfare and Ethical Review Body and experiments carried out during day time according to the UK Home Office License regulations.

Animals were immunized by the intramuscular route with two NIBRG-14 virosomal vaccine doses containing 1.5, 7.5 or 30μg HA, 14 days apart, (n = 10) formulated with 50μg Matrix M or vaccine without the adjuvant (30μg HA, n = 10). Control animals received PBS (n = 10). Animals were bled from the jugular vein pre vaccination and 12 days after each immunization. Serum samples were stored at -20°C until testing in the serological assays. Fourteen days after the second immunization, animals were sedated with 0.2mL/kg of Ketamine/Xylazine and challenged by intranasally administering A/Vietnam/1203/2004 live virus (10^6^ egg infectious dose (EID)_50_ in 0.4mL PBS/BSA). Following challenge all animals were monitored for fever and weight change.

Three days post-challenge, four animals from each group were sacrificed and the lungs, nasal turbinates, brains, olfactory bulbs and spleens were collected. The remaining animals were observed for clinical signs of disease until 14 days post- challenge, when surviving animals were exsanguinated under terminal anaesthesia. Animals whose condition reached the moderate severity limit of the study were exsanguinated under terminal anaesthesia. The challenge study was conducted under enhanced BSL-3 conditions.

### Virus recovery from nasal washings and tissue samples from ferrets

Immediately after collection, ferret tissues were weighed and homogenized in L15 medium containing penicillin (1000 IU) and streptomycin (100μg/ml). Homogenates were clarified by centrifugation and aliquots frozen at -70°C. Nasal washings, and clarified tissue homogenates were inoculated undiluted or diluted (10^-1^–10^-8^) into Madin-Darby Canine Kidney (MDCK) cells in 96 well tissue culture plates and incubated at 35°C for three days. Aliquots of medium from each well were transferred to wells of U-well microtitre plates and the presence of replicating virus detected using 0.7% turkey blood. Virus titres were calculated by the method of Spearman-Karber [16]. The limit of detection in Tissue Culture Infectious Dose (TCID)_50_ assay for the ferret tissue samples was 10^1.63^ TCID_50_/ml of homogenate.

### Statistics

Statistical analysis was performed by GraphPad Prism version 6 for Mac OS X (GraphPad Software, Ja Jolla, CA, USA) and the longitudinal data were analyzed by R 3.1.1 (R Core team). In the human clinical trial, the MN, SRH, HI and serum IgG titres and ASC and B_mem_ responses between the virosomal alone and adjuvanted vaccine groups were assessed by ANOVA followed by Dunnetts multiple comparisons test. The longitudinal serological and ELISA data were analyzed using a linear mixed model (assuming compound symmetry) with the unadjuvanted (30-) dose a reference and time as a continuous variable. The longitudinal data were analyzed only up to day 42 post-vaccination as too few sample were available beyond this time point. Spearman rank correlation coefficient (r) values (adjusted for multiple comparisons) were calculated to determine the correlation between the NIBRG-14-specific and cross-H5 clade serological responses. In the ferret challenge model, the virus titres recovered from nasal washings and tissue samples and body temperatures between the vaccinated and control groups were assesses by ANOVA (with Dunnett’s multiple comparisons test). *p*-values <0.05 were regarded as significant in all statistical tests.

## Results

**Fig 1:**
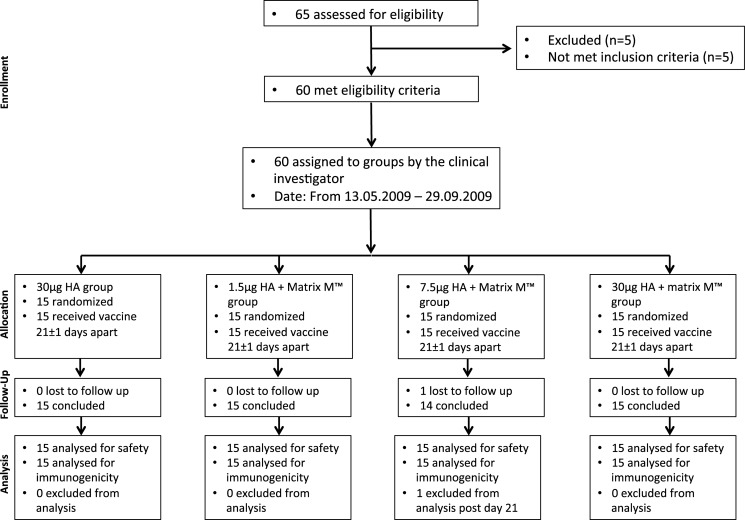
CONSORT flowchart. In total, 65 adult subjects were assessed for eligibility and 60 were included in the study (mean age 31 years old, 60% female). Prior to being enrolled in the study, all participants provided written informed consent and were screened for normal haematology, biochemistry and immunology parameters. The 60 volunteers were randomised into four groups and were immunised twice, 21 days (±1) apart with the virosomal H5N1 vaccine alone (30μg HA) or with vaccine (1.5, 7.5 or 30μg HA) formulated with Matrix-M (50μg) adjuvant. Blood samples were collected at 7, 14 and 21 days after each dose of vaccine and 180 and 365 days after the first vaccination.

In this study, we investigated the early kinetics and the long-term persistence of the serological responses induced by a candidate H5N1 vaccine formulated with or without the Matrix M adjuvant. Serological responses were determined against both the homologous vaccine as well as clade 1 and 2 H5N1 strains. Fig 1 shows the CONSORT flowchart of the clinical trial and the CONSORT checklist (S1 File), N3CRs Arrive guidelines (S2 File) and study protocol (S3 File) are provided as supporting information. The protective efficacy of the vaccine formulations against challenge with HPAI virus was tested in a ferret infection model.

### Microneutralization response to vaccination

**Fig 2:**
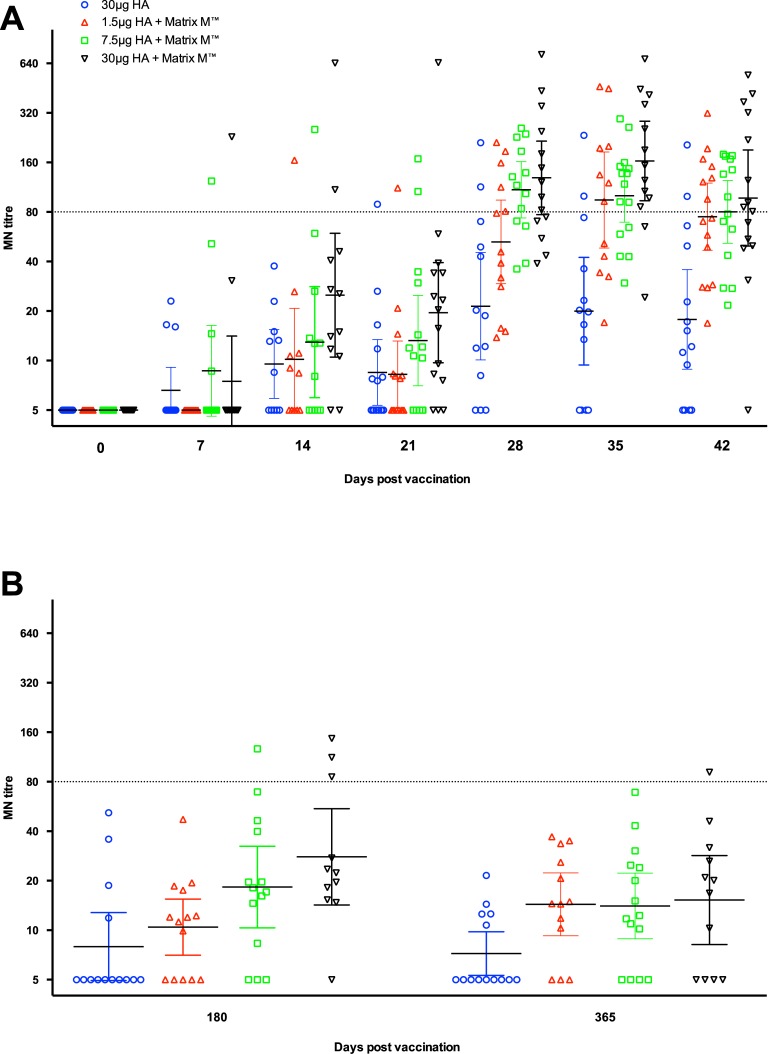
The kinetics and long-term microneutralization response after vaccination. The kinetics (A) and long-term (B) microneutralization (MN) antibody response to the homologous vaccine strain A/Vietnam/1194/2004 (NIBRG-14) after vaccination with two doses (21 ±1 days apart) of inactivated virosomal H5N1 vaccine alone (30μg HA, blue) or 1.5 (red), 7.5 (green) or 30μg HA (black) adjuvanted with Matrix-M (50μg). The sampling day after vaccination is shown on the x-axis. Each symbol represents the geometric mean MN titre for one individual participant, with the group geometric mean titre and 95% confidence interval presented. The dotted line indicates the protective MN titre of 80.

Fig 2 shows the kinetics (Fig 2A) and long-term (Fig 2B) MN responses in humans after vaccination. A seroprotective MN response was defined as a MN titre ≥80 [17]. No pre-vaccination neutralizing antibodies were detected in any of the subjects. MN responses were detectable by day 7 in all groups (except the adjuvanted 1.5μg HA group) with one subject each from the adjuvanted 7.5μg and 30μg HA groups having a protective antibody response. At day 14, higher MN geometric mean titres (GTMT) were observed in the adjuvanted 30μg HA (GMT = 25) and 7.5μg HA (GMT = 13) groups compared with the virosomal alone group (GMT = 9), however, when considering only the responding subjects, the differences were not statistically significant. The response plateaued to day 21 with at least one subject from each vaccine group having protective neutralizing responses. The second immunization augmented the MN response in all vaccine groups. On day 28, 38% of vaccinees in the 1.5μg HA group and 64% in the adjuvanted 30μg HA group had MN titres ≥80. In comparison, only 15% of volunteers in the virosomal alone group had protective MN titres at day 28. By day 35, an increase in the MN GMTs was observed in the adjuvanted 1.5μg HA (GMT = 95, 58% seroprotected) and 30μg HA (GMT = 163, 85% seroprotected) groups while titres slightly decreased in the other two vaccine groups. When considering only the responding subjects, the adjuvanted 30μg HA group had significantly higher GMTs compared with the non-adjuvanted group at days 28, 35 and 42 post-vaccination. On day 42, a dose-dependent response was observed in the adjuvant groups with between 46% and 60% of subjects having protective neutralizing antibody titres, whilst only 14% in the virosomal alone group were seroprotected.

Fig 2B shows the long-term NIBRG-14-specific MN responses after vaccination. At 6 months post-vaccination (day 180), only three persons in the adjuvanted 30μg HA group and one from the 7.5μg HA group had protective titres while none from the 1.5μg HA or virosomal alone groups had seroprotective titres. An antibody response was detected in 64% to 90% of subjects given an adjuvanted vaccine while only 29% of volunteers in the unadjuvanted vaccine group had a detectable antibody response. At 12 months post-vaccination, one person in the adjuvanted 30μg HA group had a protective MN response and antibody was still detectable in 66% to 77% of the subjects vaccinated with an adjuvanted vaccine and in 35% of subjects in the unadjuvanted vaccine group.

### Single radial haemolysis antibody response to vaccination

**Fig 3:**
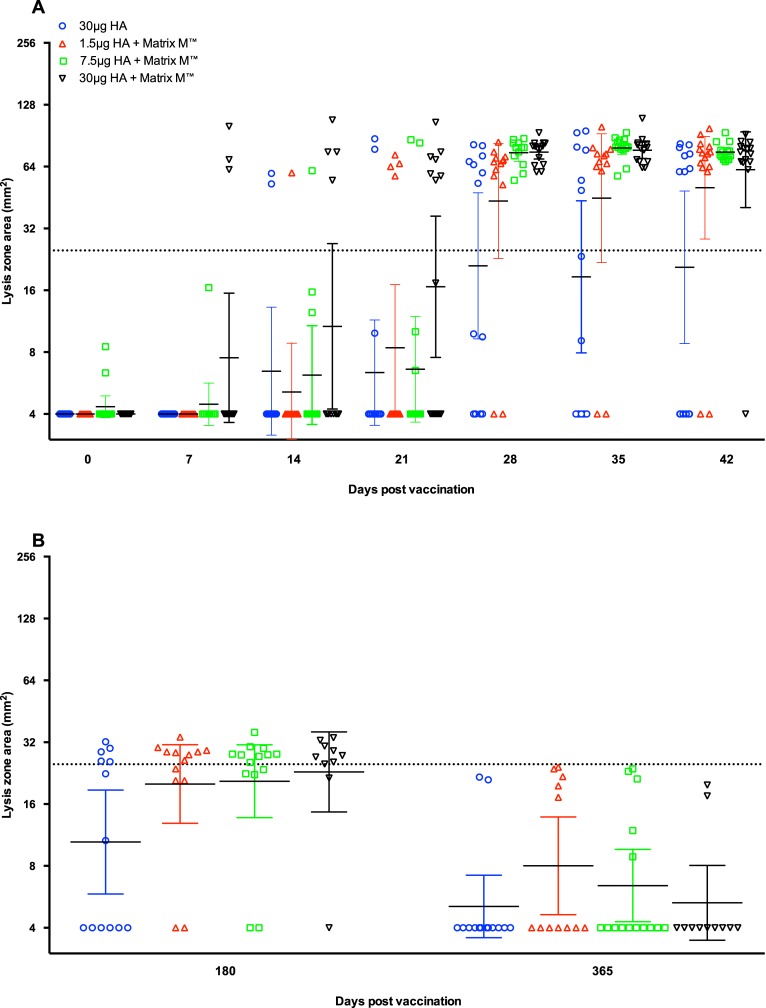
The kinetics and long-term single radial haemolysis antibody response induced after vaccination. The kinetics (A) and long-term (B) single radial haemolysis response to the homologous vaccine strain A/Vietnam/1194/2004 (NIBRG-14) after vaccination with two doses (21 ±1 days apart) of inactivated virosomal H5N1 vaccine alone (30μg HA, blue) or 1.5 (red), 7.5 (green) or 30 μg HA (black) adjuvanted with Matrix-M (50μg). The sampling day after vaccination is shown on the x-axis. Each symbol represents the geometric mean lysis zone area (mm^2^) for one individual participant, with the group geometric mean and 95% confidence interval presented. The dotted line shows the seroprotective SRH Zone area of ≥25mm^2^.

Fig 3A shows the kinetics of the SRH response to the homologous vaccine strain NIBRG-14. A seroprotective SRH response was defined as a SRH zone area ≥25mm^2^. No SRH antibodies were detected pre vaccination apart from two volunteers in the 7.5 μg HA adjuvanted group who had very low SRH zone areas of 6 and 9mm^2^. The SRH responses started to increase from day 7 post-first vaccination with 3 out of 15 subjects in the 30μg HA adjuvanted group having SRH zone areas ≥25 mm^2^. The SRH titres continued to increase by day 14-post first vaccination, with 4 subjects in the 30μg HA adjuvanted group and 1–2 subjects in other groups having SRH zone areas ≥25 mm^2^. By day 21, 7 of 15 subjects (47%) in the adjuvanted 30μg HA group had SRH zone areas ≥25 mm^2^, whereas only 2 to 4 vaccinees had protective antibody titres in the other vaccine groups. The second immunization augmented the SRH titres in all of the vaccine and by day 28, all of the subjects in the adjuvanted 7.5μg HA and 30 μg HA groups had SRH zone areas ≥25 mm^2^, whilst high SRH titres were also detected in the adjuvanted 1.5μg HA (85% protected) and virosomal alone groups (54% protected). When considering only the responding subjects, the adjuvanted 7.5μg HA and 30 μg HA groups had significantly higher GMT zone areas compared with the non-adjuvanted group at day 28. Similarly at day 35, the 7.5μg HA group had significantly higher SRH zone areas compared with the non-adjuvanted group. Among the volunteers that received an adjuvanted vaccine, between 0% and 13% failed to elicit a SRH response at day 42, whilst the number of non-responders was 43% in the non-adjuvanted group.

Fig 3B shows the SRH titres at 6 and 12 months post-vaccination. At 6 months post-vaccination, a majority of subjects (61% to 80%) who had received an adjuvanted vaccine had SRH zone area ≥25mm^2^, whilst 38% of volunteers in the virosomal alone group had protective SRH titres. The response decreased by 12 months post-vaccination where SRH titres could only be detected in 12% to 42% of subjects, although none of the volunteers had titres above the protective threshold.

### The kinetics and cross-reactivity of the haemagglutination inhibition antibody response to vaccination

**Fig 4:**
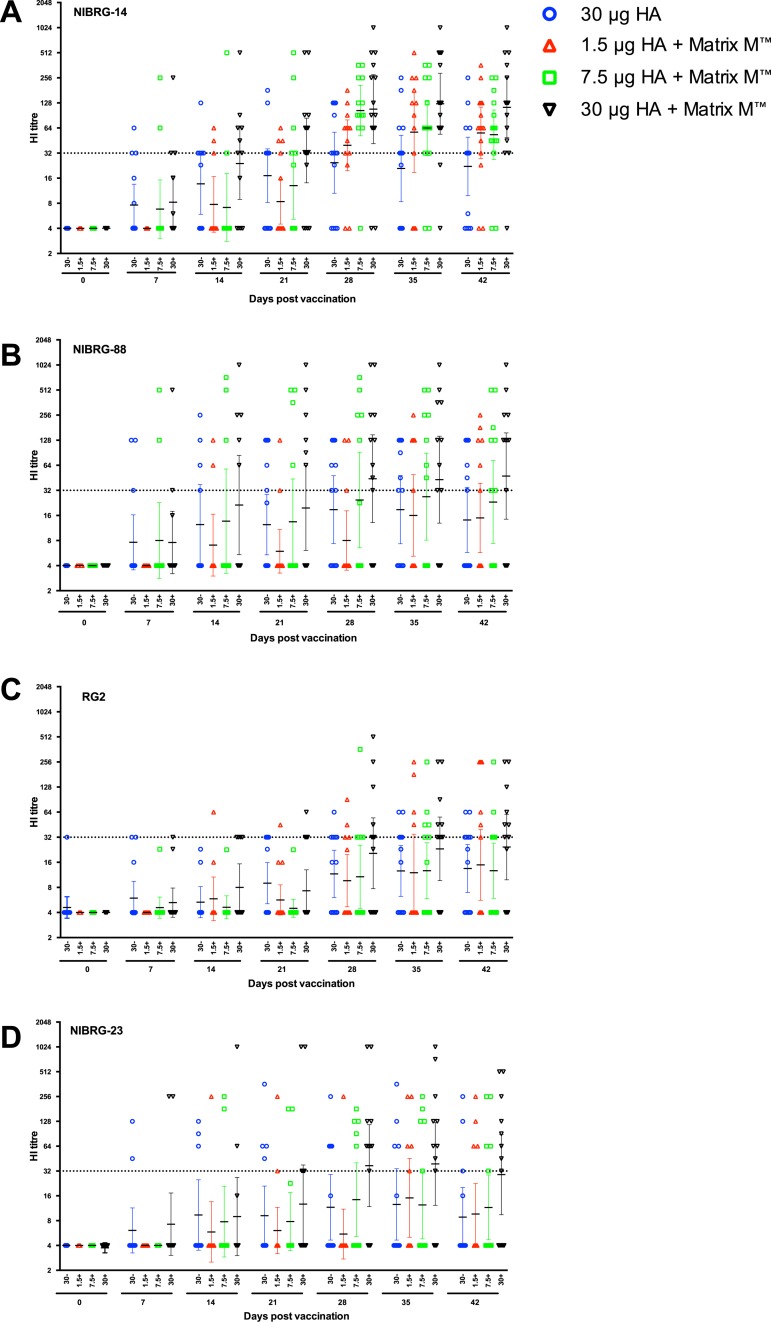
The kinetics of the haemagglutination inhibition antibody response induced after vaccination. The kinetics of the haemagglutination inhibition (HI) antibody response to the homologous vaccine strain (A) A/Vietnam/1194/2004 (NIBRG-14) and the cross-H5 clade HI responses to (B) A/Cambodia/R0405050/2007 (NIBRG-88), (C) A/Indonesia/5/2005 (RG2) and (D) A/turkey/Turkey/1/2005 (NIBRG-23) strains were measured by the HI assay. Subjects were vaccinated with two doses (21 ±1 days apart) of inactivated virosomal H5N1 vaccine alone (30μg HA, blue) or 1.5 (red), 7.5 (green) or 30μg HA (black) adjuvanted with Matrix-M (50μg). The sampling day after vaccination is shown on the x-axis. The HI titres were measured by the modified HI test using horse erythrocytes. Each symbol represents the geometric HI titre for one individual, with the group geometric mean and 95% confidence interval presented. The dotted line shows the protective HI titre of 32.

The kinetics of the HI response against the homologous vaccine strain (NIBRG-14) following vaccination is shown in Fig 4A. A seroprotective response was defined as an HI titre ≥32. No pre vaccination HI antibody titres were detected in any of the volunteers. The NIBRG-14-specific HI antibody titres started to increase at 7 days post vaccination in all groups, except the lowest adjuvanted dose. By day 14, an increase in the NIBRG-14-specific HI response was detected in all vaccine groups and the titres continued to increase up to day 21. On day 21, a majority of subjects (66%) in the 30μg HA adjuvanted group (GMT = 34) were seroprotected. The second vaccine dose enhanced the NIBRG-14-specific HI responses in all vaccine groups. Among the responding subjects, significantly higher HI GMTs were observed in the adjuvanted 30μg HA group compared with the non-adjuvanted group at days 28 and 42 post-vaccination. A majority of subjects immunized with one of the adjuvanted vaccines (75% to 93%) had seroprotective HI response after the second vaccination (between days 28 and 35), whilst during the same period, between 54% and 64% in the vaccine alone group were seroprotected. By day 42, similar GMTs were observed in the 1.5μg (GMT = 56) and 7.5μg (GMT = 53) HA adjuvanted groups showing the potential of Matrix M adjuvant to dose spare.

The HI assay was used to examine the kinetics of the post-vaccination response to three other H5N1 strains; clade 1 virus A/Cambodia/R0405050/2007 (NIBRG-88, Fig 4B, clade 1.1), and clade 2 viruses A/Indonesia/5/2005 (RG2, clade 2.1.3.2, Fig 4C) and A/turkey/Turkey/1/05 (NIBRG-23, clade 2.2.1 Fig 4D). No cross-reactive pre-vaccination HI antibodies were detected in any of the volunteers, except to A/Indonesia/5/2005 where one subject in the 30μg HA virosomal group had a pre vaccination antibody titre of 32 which did not boost after vaccination. Following the first dose, the strongest cross-reactive HI response was observed against the NIBRG-88 strain, with HI titres peaking at 14 days post-vaccination in all four vaccinated groups, which then plateaued until day 21. By day 21 post-vaccination, 13% to 40% of vaccinees were seroprotected against NIBRG-88 and most of these were in the 30μg HA adjuvanted group. A similar response was observed against the NIBRG-23 strain with 13% to 35% of volunteers having protective HI titres at day 21 post-vaccination and most of these were in either the 30μg HA adjuvanted or virosomal alone groups. After the first dose, the lowest HI titres were detected against the RG2 strain with only 10 volunteers (across all four vaccine groups) having HI antibody titres ≥32 at day 21 post-vaccination. The second vaccine dose augmented the cross-reactive response and it generally peaked on day 35 with 64% of volunteers in the 30μg HA group having protective responses against NIBRG-23 (GMT = 39), NIBRG-88 (GMT = 43) and RG2 (GMT = 29) strains. In contrast, at day 35, only 23% to 54% of the subjects in the non-adjuvanted group had protective titres against NIBRG-23 (GMT = 13), NIBRG-88 (GMT = 19) and RG2 (GMT = 13). The day 42 HI responses were similar to that observed on day 35 with 57%-64% of volunteers in the adjuvanted 30μg HA group having seroprotection against NIBRG-88 (GMT = 43), NIBRG-23 (GMT = 29) and RG2 (GMT = 23). In contrast, only 14 to 43% of volunteers in the virosomal alone group had protective responses against NIBRG-88 (GMT = 14), NIBRG-23 (GMT = 9) and RG2 (GMT = 13) at day 42. Importantly, after two doses of the vaccine (day 42), the number of subjects who failed to induce a cross-H5 clade HI response (non-responders) was lower in the adjuvanted 30μg HA group (26% to 36%) compared with the non-adjuvanted vaccine (43% to 64%).

### Longevity of the haemagglutination inhibition response to vaccination

**Fig 5:**
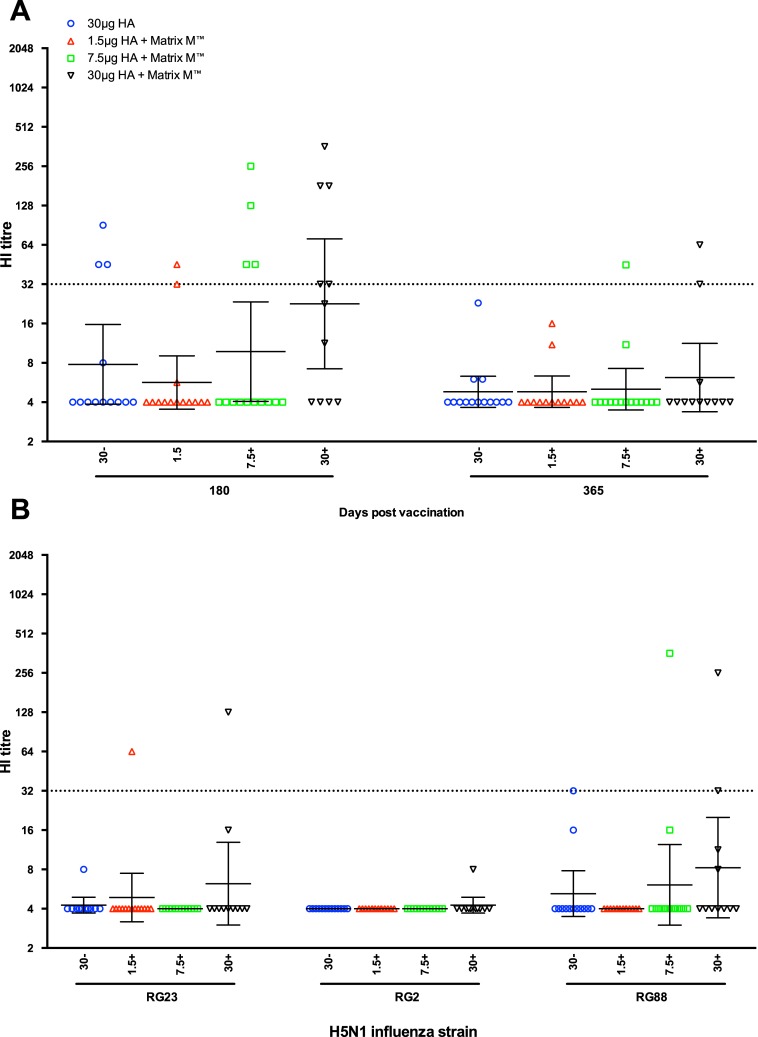
The longevity of the haemagglutination inhibition antibody response induced after vaccination. A: The haemagglutination inhibition (HI) response against the homologous vaccine strain A/Vietnam/1194/2004 (NIBRG-14) at 180 and 365 days after vaccination. B: The cross-H5 clade HI response to A/turkey/Turkey/1/2005 (NIBRG-23), A/Indonesia/5/2005 (RG2) and A/Cambodia/R0405050/2007 (NIBRG-88) at 180 days post-vaccination. Subjects were vaccinated with two doses (21 ±1 days apart) of inactivated virosomal H5N1 vaccine alone (30μg HA, blue) or 1.5 (red), 7.5 (green) or 30 μg HA (black) adjuvanted with Matrix-M (50μg). The HI titres were measured by the modified HI test using horse erythrocytes. Each symbol represents the geometric HI titre for one individual participant, with geometric mean titres for the group and 95% confidence interval presented. The dotted line shows the protective HI titre of 32.

Fig 5A shows the NIBRG-14-specific HI responses at days 180 and 365 post-vaccination. On day 180, the 45% of subjects in the adjuvanted 30μg HA (GMT = 23) and 29% in the 7.5μg HA (GMT = 10) groups were seroprotected, whilst in the 1.5μg HA group, two persons (21%) had a seroprotective response (GMT = 6). In the virosomal alone vaccine group, 23% of subjects had HI titres ≥32 (GMT = 8). At 12 months after vaccination, only two volunteers from the adjuvanted 30μg HA group and one from the 7.5μg HA had seroprotective HI titres. Fig 5B shows that generally no long term protective HI responses were observed against the other H5N1 strains, except four persons (across all four vaccinated groups) were seroprotected against NIBRG-88 and two against NIBRG-23 strains at 6 months post vaccination. No cross-H5 clade HI responses were observed at 12 months post-vaccination (data not shown).

### The H5N1-specific serum IgG response

**Fig 6:**
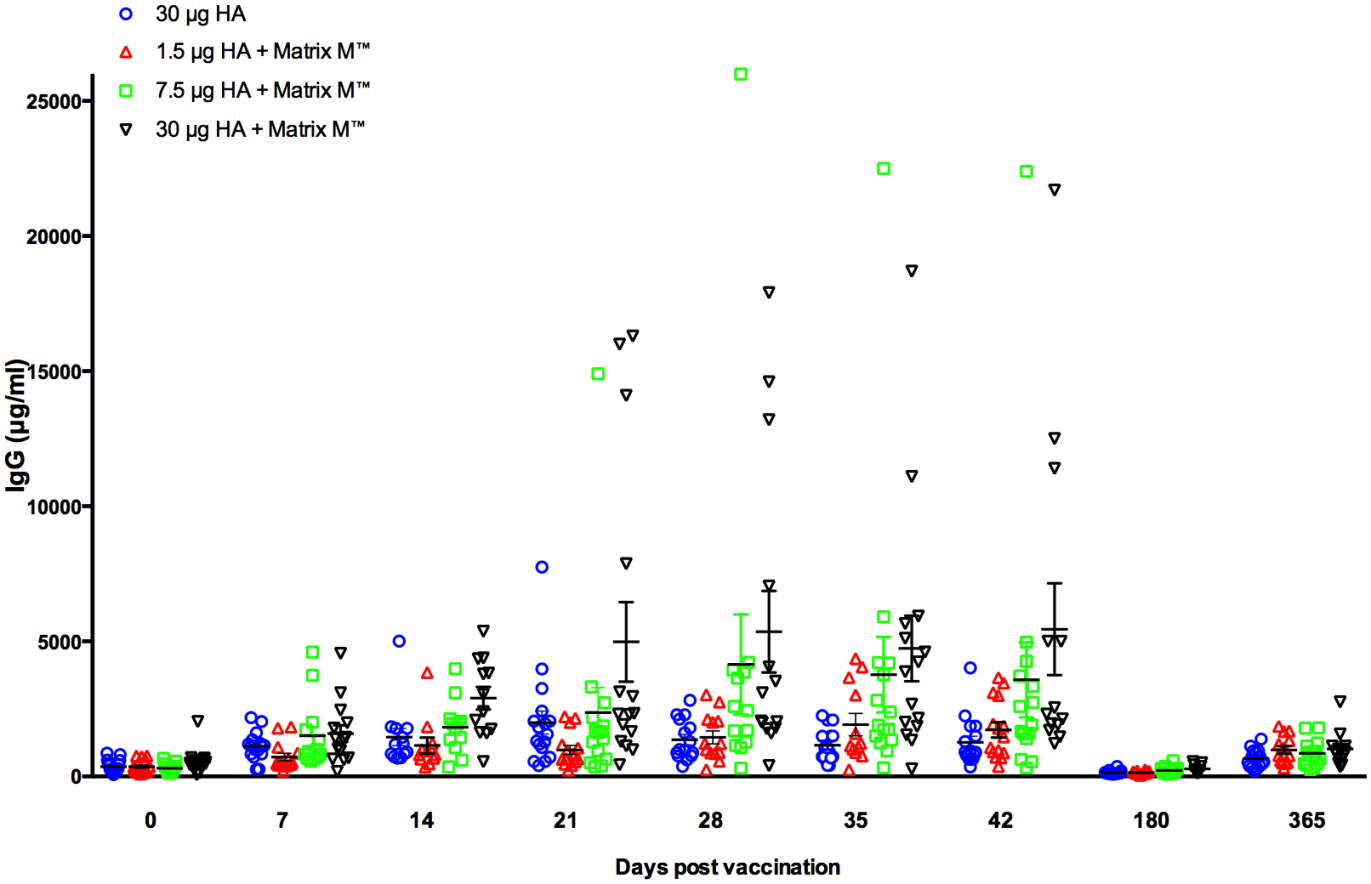
The H5N1-specific IgG response after vaccination. An ELISA was used to measure the NIBRG-14 influenza vaccine-specific serum IgG response before and after vaccination. Subjects were vaccinated with two doses (21 ±1 days apart) of inactivated virosomal H5N1 vaccine alone (30μg HA, blue) or 1.5 (red), 7.5 (green) or 30μg HA (black) adjuvanted with Matrix-M (50μg). The bars represent the mean IgG titre ± standard error of the mean.

Fig 6 shows the serum NIBRG-14-specific IgG response after vaccination. Very low NIBRG-14-specific antibody levels were observed prior to vaccination.

In comparison to day 0, an increase in the IgG response was detected in all vaccine groups by days 14 and 21, with the adjuvanted 30μg HA group having significantly higher responses than the non-adjuvanted group. The second dose (day 28) did not boost the IgG response in any group except in the adjuvanted 7.5μg HA group, however this was not statistically significant compared with day 21 levels. After the second dose, the highest IgG levels were observed in the adjuvanted 30μg HA group, which had a significantly higher IgG response than the non-adjuvanted vaccine at 28, 35 and 42 days post-vaccination. Among the adjuvanted groups, a dose response was observed with significantly higher IgG levels detected in the adjuvanted 30μg HA group compared to that in the 1.5μg HA group after the first and second vaccinations. At six and 12 months post-vaccination, the serum IgG levels in each of the vaccine groups were significantly lower compared with that at day 42.

### The correlation between homologous and cross-H5 clade serological responses

**Fig 7:**
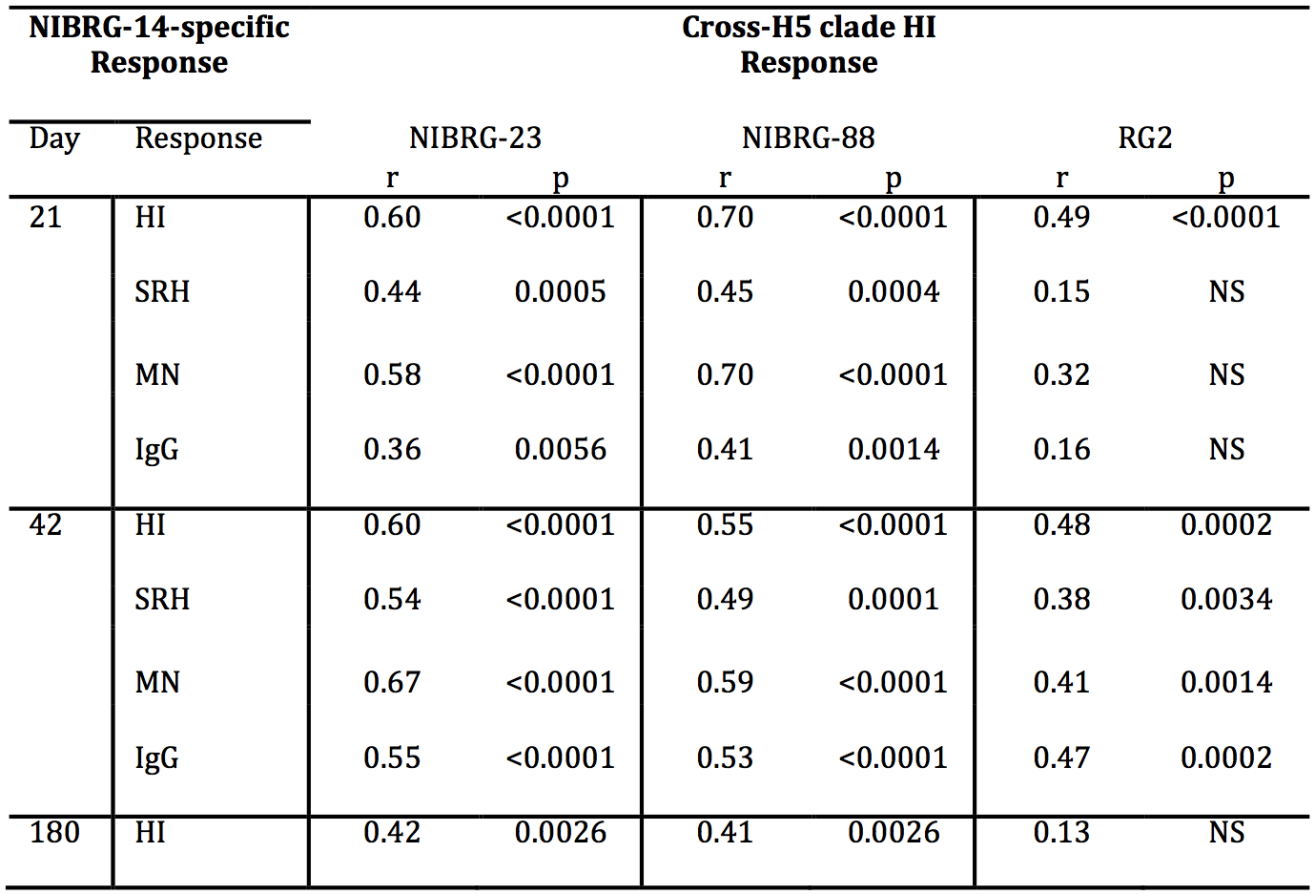
Correlation between serological responses to the homologous and cross-reactive H5N1 strains at days 21, 42 and 180 after vaccination. The correlation between A/Vietnam/1194/2004 (NIBRG-14)-specific HI, SRH, MN and IgG responses and the HI responses against A/turkey/Turkey/1/2005 (NIBRG-23), A/Cambodia/R0405050/2007 (NIBRG-88) and A/Indonesia/5/2005 (RG2) at 21, 42 and 180 days after vaccination. Volunteers were divided into four vaccine groups and were vaccinated with two doses (21 ±1 days apart) of inactivated virosomal H5N1 vaccine alone (30μg HA) or 1.5, 7.5 or 30μg HA adjuvanted with Matrix-M (50μg). Data from the four vaccine groups were combined to calculate the Spearman rank correlation coefficient (r) value (adjusted for multiple comparisons) for each association between the homologous and cross-H5 clade serological response. Abbreviations: p, Two-tailed p value (95% confidence interval); NS, no significant correlation.

To test whether subjects who had high NIBRG-14-specific serological responses (HI, SRH, MN and serum IgG) also had good cross-H5 clade HI responses, we performed Spearman rank correlation coefficient (r) analysis at 21, 42 and 180 days post-vaccination (Fig 7). Subjects that had higher NIBRG-14-specific HI, SRH, MN and IgG responses also had good HI responses to NIBRG-23 and NIBRG-88 strains with Spearman r values ranging between 0.36 and 0.7. The correlation between the homologous serological responses and HI responses to the RG2 strain were less robust. On day 21, the RG2-specific HI response showed a significant correlation with the homologous HI response (r = 0.49, p<0.0001) but not with the SRH, MN or IgG responses. However, by day 42, a significant correlation was observed between the NIBRG-14-specific HI, SRH, MN and IgG responses and the RG2-specific HI responses, with Spearman r values ranging between 0.38 and 0.48. At six months post-vaccination, the NIBRG-14-specific HI responses showed significant correlation with the NIBRG-23 and NIBRG-88-specific HI responses, but not with RG2-specific HI titres.

### The antibody secreting cell response

**Fig 8:**
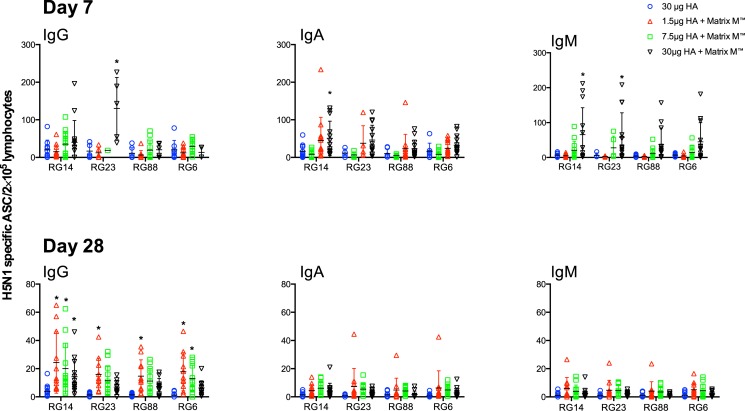
The antibody secreting cell response induced after influenza vaccination. Lymphocytes were collected 7 days after the first (day 7) and second vaccine dose (day 28) of inactivated virosomal H5N1 vaccine alone or formulated 1.5, 7.5 or 30μg HA formulated with Matrix-M (50μg). The influenza-specific IgG, IgA and IgM antibody secreting cells (ASC) were enumerated using the ELISPOT assay. The data are presented as the mean number of influenza-specific ASC per 200 000 lymphocytes ± standard error of the mean to the homologous vaccine strain A/Vietnam/1104/2004 (NIBRG-14), and the heterologous responses to A/Turkey/Turkey/1/05 (NIBRG-23), A/Cambodia/R0405050/2007 (NIBRG-88) and A/Anhui/1/05 (RG6). Statistical differences between the adjuvanted and non-adjuvanted groups were calculated by ANOVA with Dunnett’s multiple comparisons test. *p<0.05.

No or very low NIBRG-14-specific ASCs were detected prior to vaccination (data not shown). Fig 8 shows the homologous and cross-H5 clade IgG, IgA and IgM ASC responses after the first (day 7) and second (day 28) vaccine doses. On day 7, the strongest IgG, IgA and IgM ASC responses against both the homologous and cross-reactive strains were detected in the groups that received the adjuvanted high dose (7.5 and 30μg HA) vaccines. After the second dose (day 28), the strongest ASC responses were observed in the group that received the adjuvanted 1.5μg HA vaccine and the ASC responses were dominated by IgG while very low IgA and IgM levels were detected.

### Memory B cell responses

**Fig 9:**
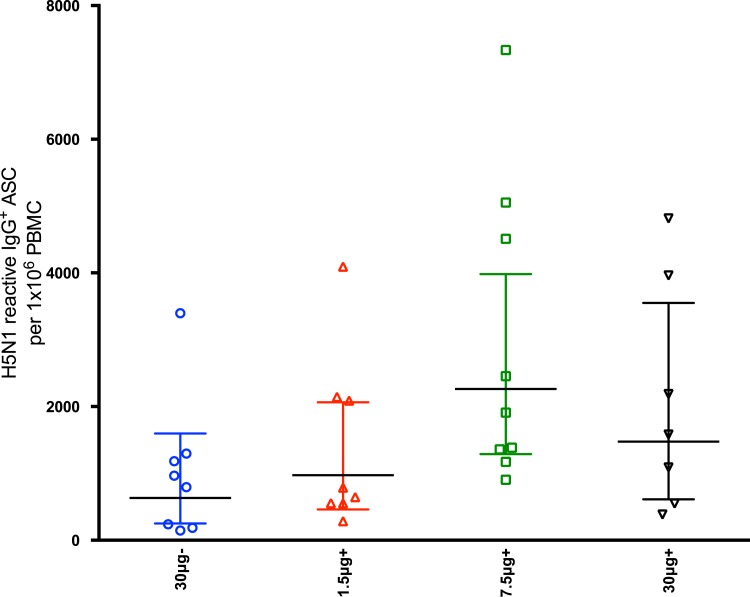
The memory B cell (B_mem_) response 365 days after vaccination. Subjects were vaccinated with two doses (21 ±1 days apart) of inactivated virosomal H5N1 vaccine alone (30μg HA, blue) or 1.5 (red), 7.5 (green) or 30μg HA (black) adjuvanted with Matrix-M (50μg). The B_mem_ response at 365 days post-first vaccination was analyzed by ELISPOT. The bars represent the mean number of NIBRG-14-specific B_mem_ per 1×10^6^ peripheral blood mononuclear cells (PBMC) ± standard error of the mean.

We evaluated the long-term homologous B_mem_ response in 7–8 randomly selected volunteers from each vaccine group at day 365 post-vaccination (Fig 9). There was a trend towards higher NIBRG-14 virosome-specific B_mem_ frequencies in subjects vaccinated with the adjuvanted 7.5 μg and 30μg HA than in virosomal alone group (mean of 2264, 1475 and 632 B_mem_ per 1 × 10^6^ PBMC, respectively).

### Protective efficacy of the H5N1 vaccine in ferrets

The protective efficacy of the NIBRG-14 virosomal vaccine was confirmed in a ferret challenge model of influenza infection. In this study, animals were vaccinated twice (14 days apart) mimicking a shorter vaccination schedule, which could be appropriate in a pandemic scenario.

**Fig 10:**
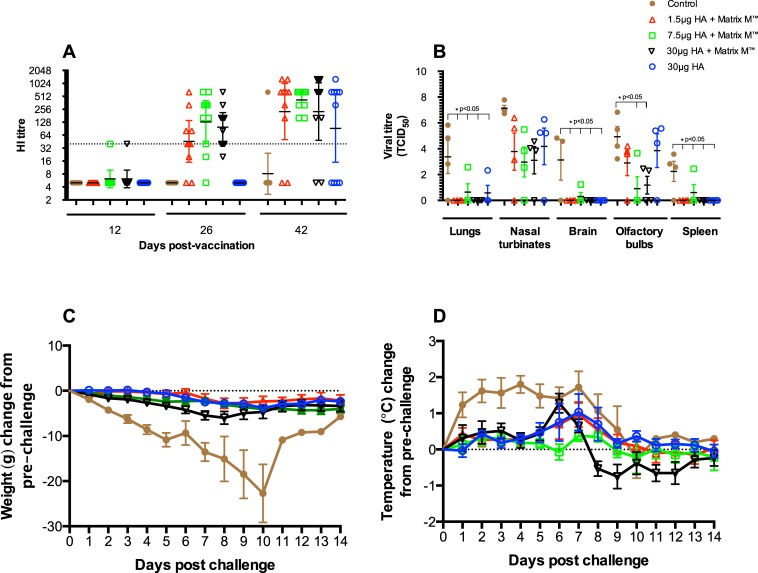
Protective efficacy of the H5N1 vaccine in ferrets. Ferrets were divided into five groups of 10 and were vaccinated intramuscularly with two doses (14 days apart) of virosomal vaccine alone (30μg HA, blue) or 1.5 (red), 7.5 (green) and 30μg HA (black) adjuvanted with Matrix M (50μg). Control ferrets (brown) received PBS. Fourteen days after the second immunization, ferrets were challenged by administering A/Vietnam/1203/2004 live virus (10^6^ EID_50_ in 0.4 mL PBS/BSA). (A) The serum HI antibody response 12 days after each vaccination with NIBRG-14 virosomal vaccine and fourteen days following challenge with A/Vietnam/1203/2004 virus. (B) The virus titres in the nasal washing and tissue samples after challenge with the A/Vietnam/1203/2004 virus. The limit of detection in TCID_50_ assay for the ferret tissue samples was 10^1.63^TCID_50_/ml of homogenate. (C) The changes in weight and (D) temperature after challenge with A/Vietnam/1203/2004 live virus.

Following the first vaccination, HI titres ≥40 were detected in one animal each from the adjuvanted 7.5 and 30μg HA groups (Fig 10A). After the second dose, the majority of ferrets (70–90%) in the adjuvanted vaccine groups had HI titres ≥40, whilst no HI responses were observed in the unadjuvanted vaccine or control groups. After the virus challenge, 80–100% of animals in the adjuvanted vaccine and 60% in the unadjuvanted vaccine groups had HI titres above the protective threshold.

Three days after the virus challenge, 4 animals from each group were sacrificed and the lung, nasal turbinate, brain, olfactory bulb and spleen samples were collected and viral titres determined (Fig 10B). Significantly low mean viral loads were detected in the lung, brain and spleen samples obtained from vaccinated ferrets compared with that from control animals. Lower mean virus titres were also detected in the nasal turbinate samples from vaccinated animals compared with controls although this difference was not statistically significant. In the olfactory bulb samples, significantly lower mean virus titres were detected in ferrets vaccinated with the adjuvanted 7.5 and 30μg HA vaccines compared with control animals.

The remaining 6 ferrets in each group were monitored daily for 14 days for body weight and clinical signs of disease post-challenge. Fig 10C shows the body weight of the ferrets was monitored for 14 days post viral challenge. In vaccinated ferrets, the body weights remained stable with minor fluctuations during the two-week period and all the animals survived the virus challenge. In the control group, the body weights decreased from day 2 with only 1 animal surviving the virus challenge to 14 days post-challenge. Control ferrets were sacrificed in a moribund condition (2 animals each on days 5 and 6, 1 ferret on day 10) due to signs of severe clinical infection and/or excessive weight loss.

Fig 9D shows the body temperatures post-virus challenge. All the control animals developed fever (temperature rise >1°C) within 1–2 days after viral challenge. Ferrets that received the adjuvanted 7.5μg HA vaccine did not develop fever during the two-week period. Ferrets vaccinated with the adjuvanted 1.5μg HA and non-adjuvanted vaccines developed fever one week post-challenge, but the temperatures returned to baseline levels after two weeks. Greater temperature fluctuations were observed in ferrets that received the adjuvanted 30μg HA vaccine, however the temperature returned to pre-challenge levels two weeks after vaccination. Statistical analysis of the temperatures five days after challenge, which was the last time point at which all the animals in the control group were alive, showed that the mean body temperatures in the adjuvanted 7.5 μg and 30μg HA vaccine groups were significantly lower than that of control group. Furthermore, the maximum observed temperature increase in the 7.5μg HA group was significantly lower than for the control group (p<0.004).

## Discussion

Correlates of protection against avian influenza are not established and there is a need for more detailed analysis of the kinetics, quality and magnitude of the immune response after candidate pandemic vaccination. In this study, we report in detail the early kinetics and the long-term persistence of the homologous and cross-reactive immune responses after vaccination with a candidate H5N1 influenza vaccine formulated with or without the Matrix M adjuvant. Overall, analysis of the longitudinal data using a mixed model shows that formulation with the Matrix M adjuvant significantly enhanced (P<0.05) the serological (HI, SRH, MN) and IgG responses against H5N1 influenza viruses (S1 Table).

Vaccination is still the best means of combating an eventual pandemic caused by a HPAI H5N1 virus. Our findings, using the three commonly used serological assays (HI, SRH and MN) revealed a low antibody response against NIBRG-14 after the first vaccination (days 7 and 14), with only a few subjects mainly in the adjuvanted 7.5 and 30μg HA groups having protective responses. Three weeks after the first dose, 66% and 47% of subjects in the 30μg adjuvanted group had protective titres by HI and SRH assays, respectively, however only 7% had protective MN titres. This may reflect the different sensitivities of the serological assays and is in contrast to previous studies where higher sensitivities were reported for MN compared to the HI assay (reviewed by [18]). Our HI titres are comparable with that observed after a single dose of H5 vaccines adjuvanted with oil-in-water adjuvants, but higher than HI titres elicited by alum-adjuvanted H5 vaccines [11, 19–21]. This is consistent with observations from a number of clinical trials where alum failed to greatly improve the immune responses to split virus influenza vaccines in humans (reviewed by [3]).

The second vaccine dose greatly improved the protective antibody responses to the NIBRG-14 virus as measured by the HI, SRH and MN assays, but no significant increase was detected in the H5N1 specific serum IgG response. The day 42 NIBRG-14-specific serological responses, which were strongest in the adjuvanted 30μg HA group, were comparable to those observed in adult subjects after two doses of oil-in-water adjuvanted H5 vaccines [11, 19, 22, 23], but substantially higher than an alum adjuvanted vaccine [20] or a high dose (90μg HA) non-adjuvanted A/Vietnam/1203/2004 vaccine [24]. These results highlight the need for appropriate adjuvant formulation of H5 influenza vaccines for enhancing immunogenicity and the superiority of the Matrix M adjuvant over more traditional adjuvants such as alum. The seroprotection rates fell significantly by six months, although the adjuvant appeared to extend the longevity of the protective responses in some subjects, as was the case in other H5N1 influenza vaccine trials [11, 20]. By 12 months after primary vaccination, almost all subjects had NIBRG-14-speciific HI, SRH and MN titres below the protective thresholds. Interestingly, we detected long-term H5N1-reactive B_mem_ cells in all vaccine groups at day 365. B_mem_ cells can be rapidly reactivated upon infection or secondary immunization and differentiate into plasma cells producing high avidity antibodies [25]. B_mem_ cells may also recognize viral escape mutants more effectively than long-lived plasma cells [26]. Maintenance of serological memory plays an important role in combating secondary and tertiary waves of disease that may occur during a pandemic.

The humoral immune response was further characterized by determining the kinetics of the serum IgG (ELISA) and ASC (ELISPOT) responses. The NIBRG-14-specific serum IgG response peaked after the first dose (day 21), but by 6 months, the titres were similar to pre-vaccination levels. Despite the low long-term IgG titres, we have recently observed that the H5 HA1-specific IgG avidity continues to rise and was highest at 6–12 months post-vaccination [27]. The NIBRG-14-specific ASC response in all groups, except in subjects vaccinated with 1.5μg HA peaked at day 7, which has previously been shown to correspond with the peak plasmablast (CD19^+^CD20^-^CD27^high^CD38^high^) response [14]. Although we did not sample a time point before day 7, we believe this to be the peak response based on our previous observations with trivalent seasonal [13], pandemic H1N1 [28] and candidate H7N1 [29] influenza vaccine studies in humans. The NIBRG-14-specific IgG ASC response in the 1.5μg HA group peaked at day 28, however as we only sampled day 28, we cannot rule out an earlier peak after the second dose. The magnitude of the day 7 NIBRG-14-specific IgG and IgA ASC response is similar to the response detected after vaccination with an alum-adjuvanted H7N1 vaccine, but substantially lower than observed after seasonal influenza vaccination in primed adults [13, 29, 30]. The weak NIBRG-14-specific ASC and serological responses (HI, SRH and MN) at day 7 may be explained by the fact that humans are generally not primed by previous exposure to avian H5N1 viruses. Therefore naïve subjects may only have a small repertoire of cross-reactive memory B cell pool that can be rapidly reactivated after vaccination. Interestingly, a relatively strong NIBRG-23-specific IgG ASC response at day 7 may suggest a memory response against this strain. Furthermore, the dose-dependent increase in IgM ASC responses (day 7) observed in groups given an adjuvanted vaccine also suggests activation of a naïve B cell response against the H5N1 viruses after vaccination.

H5N1 viruses continue to circulate in wild birds on three continents and based on phylogenetic characterization and sequence homology in the H5 HA gene, a number of distinctive H5N1 virus subgroups (clades) have been identified [31, 32]. The wide geographical spread and the considerable antigenic diversity of H5N1 viruses are significant challenges for pandemic preparedness, as effective vaccines need to elicit broadly cross-reactive antibody responses to heterologous strains. Our data demonstrated a significant HI response against the NIBRG-88 (clade 1.1) and NIBRG-23 (clade 2.2.1) strains in the group that received the adjuvanted 30ug HA vaccine. Similar improvements in cross-clade antibody responses have been observed with H5N1 vaccines adjuvanted with MF59 and AS03 compared with non-adjuvanted formulations [33, 34]. Together with clade 1 (NIBRG-14), the 1.1 and 2.2.1 phylogenetic sub-clades have caused human infections or are in current circulation, hence induction of immunity against these strains is highly relevant. The weakest cross-reactive HI response was observed against the RG-2 strain (clade 2.1.3.2) and this may reflect greater differences in the sequence homology between the HA of NIBRG-14 and RG-2 strains. Importantly, the peak cross-reactive HI responses were observed one week after the second dose. Induction of an early cross-reactive response is important, as we cannot predict which H5N1 strain will cause disease if a pandemic emerged. The long-term (six month) antibody response was poor even after formulation with the adjuvant, which is consistent with previous observations [34]. Administering a third (booster) H5 vaccine dose may induce longer lasting cross-reactive immune response as has been shown in several studies (reviewed by [3]). The cross-clade response was further analysed in the ELISPOT assay to measure ASC and to our knowledge, this is the first report showing cross-H5 clade ASC responses in humans after pandemic H5N1 vaccination.

Ferrets are a natural influenza host and represent a well-established model for influenza research (reviewed by [9]). The virosomal vaccine was tested in ferrets to investigate the number of doses and time required for inducing a protective immune response against HPAI H5N1 challenge. Vaccination reduced febrile responses, severe weight loss and prevented the local and systemic spread of the virus. Similarly, recent studies have shown that two doses of AS03 or MF59-adjuvanted H5N1 vaccines induce protective immune responses in ferrets and reduce virus shedding in the upper respiratory tract [35, 36]. The reduced virus shedding as observed in our and other studies could be an important factor in limiting the horizontal transmission of the virus to susceptible hosts. We observed that two doses of the H5 vaccine in combination with the Matrix M adjuvant was required to induce high levels of HI antibody titres and protect the animals from morbidity and mortality associated with the highly pathogenic avian influenza virus challenge. Mathematical modeling has shown that induction of a protective immune response within two weeks after the outbreak of influenza pandemic is required for maximal reduction in viral transmission [37]. Collectively, this data suggest that ideally the population should be vaccinated twice, at least four weeks before the emerging pandemic wave in order to achieve protective immunity and minimize virus transmission.

In conclusion, we have shown that the virosomal H5N1 vaccine provides protection against highly pathogenic avian influenza virus challenge in a ferret model. In humans, the H5N1 vaccine induced an early and persistent immune response that was enhanced by the addition of the Matrix M adjuvant. Combining the H5N1 vaccine with the Matrix M adjuvant resulted in broader cross-H5 clade immune responses. Results presented in this paper add to the existing evidence [5, 7, 8, 38] that shows the great potential of the Matrix M adjuvanted virosomal H5N1 vaccine.

## Supporting Information

S1 Table Analysis of the longitudinal data. (DOCX)

S1 File CONSORT checklist. (DOC)

S2 File NC3Rs Arrive guidelines. (PDF)

S3 File Study protocol. (ZIP)
