# Supplementary material for: Matrix M H5N1 Vaccine Induces Cross-H5 Clade Humoral Immune Responses in a Randomized Clinical Trial and Provides Protection from Highly Pathogenic Influenza Challenge in Ferrets
Source: PLoS One. 2015 Jul 6;10(7):e0131652. doi: 10.1371/journal.pone.0131652 (PMC4493055; doi:10.1371/journal.pone.0131652)
Supplement: S1 Table — (DOCX) [file pone.0131652.s001.docx]

**S1 Table. Analysis of the longitudinal data**

|  | **HI - RG2** | | | **HI - NIBRG-14** | | | **HI – NIBRG-23** | | | **HI - NIBRG-88** | | | **IgG ELISA - NIBRG-14** | | | **MN – NIBRG-14** | | | **SRH – NIBRG-14** | | |
| --- | --- | --- | --- | --- | --- | --- | --- | --- | --- | --- | --- | --- | --- | --- | --- | --- | --- | --- | --- | --- | --- |
| **F-test** | **p=.0722** | | | **p=.0004** | | | **p=.0013** | | | **p=.0205** | | | **p=.0009** | | | **p < 0.0001** | | | **p=.0002** | | |
|  | **B** | **SE** | **p-value** | **B** | **SE** | **p-value** | **B** | **SE** | **p-value** | **B** | **SE** | **p-value** | **B** | **SE** | **p-value** | **B** | **SE** | **p-value** | **B** | **SE** | **p-value** |
| (Intercept) | 1.582 | 0.230 | .0000 | 1.748 | 0.255 | .0000 | 1.697 | 0.318 | .0000 | 1.770 | 0.364 | .0000 | 6.471 | 0.183 | .0000 | 1.682 | 0.209 | .0000 | 1.242 | 0.191 | .0000 |
| Dose: 1.5+ | -0.329 | 0.326 | .3172 | -0.655 | 0.362 | .0757 | -0.377 | 0.450 | .4055 | -0.548 | 0.520 | .2971 | -0.484 | 0.259 | .0671 | -0.427 | 0.295 | .1531 | -0.213 | 0.270 | .4345 |
| Dose: 7.5+ | -0.392 | 0.326 | .2338 | -0.358 | 0.361 | .3260 | -0.349 | 0.451 | .4413 | -0.135 | 0.525 | .7976 | -0.226 | 0.259 | .3863 | -0.123 | 0.296 | .6777 | -0.216 | 0.271 | .4294 |
| Dose: 30+ | -0.222 | 0.325 | .4975 | -0.067 | 0.360 | .8529 | -0.185 | 0.450 | .6819 | -0.153 | 0.517 | .7681 | 0.221 | 0.259 | .3969 | -0.034 | 0.295 | .9098 | 0.207 | 0.270 | .4459 |
| time | **0.025** | **0.006** | **.0000** | **0.040** | **0.008** | **.0000** | **0.020** | **0.007** | **.0030** | **0.030** | **0.007** | **.0001** | **0.018** | **0.005** | **.0002** | **0.034** | **0.006** | **.0000** | **0.046** | **0.006** | **.0000** |
| Dose: 1.5+ : time | 0.009 | 0.008 | .2541 | **0.035** | **0.012** | **.0032** | 0.006 | 0.009 | .5355 | 0.006 | 0.011 | .5550 | 0.017 | 0.007 | .0144 | **0.045** | **0.008** | **.0000** | **0.027** | **0.009** | **.0025** |
| Dose: 7.5+ :time | 0.008 | 0.008 | .3180 | **0.035** | **0.012** | **.0028** | 0.012 | 0.009 | .1932 | 0.014 | 0.011 | .1785 | **0.024** | **0.007** | **.0005** | **0.045** | **0.008** | **.0000** | **0.037** | **0.009** | **.0000** |
| Dose: 30+ : time | **0.021** | **0.008** | **.0097** | **0.048** | **0.012** | **.0001** | **0.034** | **0.009** | **.0003** | **0.031** | **0.011** | **.0034** | **0.024** | **0.007** | **.0005** | **0.050** | **0.008** | **.0000** | **0.028** | **0.009** | **.0016** |

F-test = overall test for interaction (time × dose)

B = regression coefficient

SE = standard error

Intercept = outcome value for Dose (non-adjuvanted 30 μg HA group) and time (Day 0)

Time = continuous time variable

Dose: 1.5+ = 1.5 μg HA vaccine adjuvanted with Matrix M

Dose: 7.5+ = 7.5 μg HA vaccine adjuvanted with Matrix M

Dose: 30+ = 30 μg HA vaccine adjuvanted with Matrix M

The hemagglutination inhibition (HI) responses against the homologous vaccine virus A/Vietnam/1194/2004 (NIBRG-14), A/Indonesia/05/2005 (RG2), A/turkey/Turkey/1/2005 (NIBRG-23) and A/Cambodia/R0405050/2007 (NIBRG-88) and the microneutralization (MN), single radial hemolysis (SRH) and serum IgG ELISA responses against the RG14 virus was analyzed using a mixed model to determine which adjuvanted group was significantly different from the non adjuvanted vaccine group. In this analysis, we assed the interaction between time (up to day 45 post-vaccination) and dose using the unadjuvanted vaccine group as a reference and time as a continuous variable. As shown in Table 1, the RG14-specific serological responses (HI, SRH, and MN) and IgG ELISA responses in the adjuvanted groups differed significantly from the non-adjuvanted (30-) group (significantly different values are highlighted in pink). With regards to the RG23, RG88 and RG6-specific HI responses; only the adjuvanted 30μg HA group differ significantly from the non-adjuvanted group (significantly different vales highlighted in pink).
