# Supplementary material for: Matrix M H5N1 Vaccine Induces Cross-H5 Clade Humoral Immune Responses in a Randomized Clinical Trial and Provides Protection from Highly Pathogenic Influenza Challenge in Ferrets
Source: PLoS One. 2015 Jul 6;10(7):e0131652. doi: 10.1371/journal.pone.0131652 (PMC4493055; doi:10.1371/journal.pone.0131652)
Supplement: S3 File — (ZIP) [file pone.0131652.s004.zip › Study protocol.pdf]

Sent REK NORD
→ 14/11/08

PROTOCOL

PREPARING FOR AN INFLUENZA PANDEMIC:

A phase I study of a Virosomal Influenza A H5N1 Vaccine in Healthy Adults

Identifying number PANFLUVAC-2008, Version 2
EUDRACT Number: 2008-006940-20

Dr. Rebecca Jane Cox

Influenza Centre

University of Bergen

Section for Microbiology and Immunology

The Gade Institute

Armauer Hansen Building

N-5021 Bergen

Norway

Tel: +47 55 97 46 68

Fax: +47 55 97 46 89

E-post: rebecca.cox@gades.uib.no

30th October 2008

Rebecca Jcox
14/11/08
Bergen

Table of contents

Table of contents **2**

List of tables and figures **5**

List of abbreviations **6**

1. General administrative information **7**
- **1. Name and address of sponsor** **7**
- **2. Name and title of person authorized to sign protocol** **7**
- **3. Name, title, address and telephone number of sponsors medical expert** **7**
- **4. Name and title of investigators responsible conducting trial** **8**
- **5. Name, title, address and telephone number of qualified physician** **8**
- **6. Name and address of collaborating laboratories** **8**

2. Background Information **10**
- **1. Name and description of investigational medicinal product** **10**
  - 1.1 The antigen 10
  - 1.2 The adjuvant 10
  - 1.3 Formulation and composition 10
- **2. A summary of findings from non clinical studies that potentially have clinical significance and from clinical trials that are relevant to the trial** **11**
- **3. Summary of the known and potential risks and benefits, if any to human subjects** ... **12**
- **4. Description of and justification for the route of administration, dosage, dosage regimen and treatment period** **12**
- **5. Statement that trial will be conducted in compliance with the protocol, GCP and the applicable regulatory requirements** **13**
- **6. Description of population to be studied** **13**
  - Inclusion criteria 13
  - Exclusion criteria 14
- **7. References to literature and data that are relevant to the trial and that provide background for the trial** **14**
  - Background 14
  - Antigenic shift and drift 15
  - Infection of man by avian influenza virus 15
  - Influenza Vaccines 16
  - Virosomal vaccine 18
  - ISCOM™ adjuvant 18
  - Safety of inactivated influenza vaccine 19
  - Kinetics of the immune response to inactivated influenza vaccine 20
  - European Union (EU) requirements for influenza vaccines 20
  - Pandemic influenza vaccines 21
  - PANFLUVAC consortium 22

4. TRIAL OBJECTIVES AND PURPOSE **23**

5 TRIAL DESIGN **24**
- **1. A specific statement of the primary endpoints and secondary endpoints to be measured by the trial** **24**
  - A description of the type/design of trial to be conducted 24

2. A description of measure taken to avoid/minimize bias 25
3. A description of the trial treatment and dosage and dosage regimen of the investigational medicinal product. 25
Study treatments 27
Test investigational medicinal products (IMPs) 27
IMP accountability 29
Preparation and Administration 30
Precautions for Use 30
Logistics 31
Labeling and Packaging 31
Shipment Conditions 31
Storage Conditions 31
Replacement Doses 32
Return of Unused Products 32
4. The expected duration of subject participation and a description of the sequence and duration of all trial periods 32
STUDY PROCEDURE 35
First visit: Day -14 (±12 days): Pre-screening 35
Second visit: Day 0: First vaccination 35
Third visit: Day 3 (±1 day) post 1st vaccination 36
Fourth visit: Day 7 (±2 day) post 1st vaccination 36
Fifth visit: Day 14 (±2 days) post 1st vaccination 37
Sixth visit: Day 21 (±4 days): Second Vaccination 37
Seventh visit: Day 3 (±1 day) post 2nd vaccination 38
Eight visit: Day 7 (±2 day) post 2nd vaccination 38
Ninth visit: Day 14 (±2 days) post 2nd vaccination 38
Tenth visit: Day 21 (±4 days) post 2nd vaccination 39
Eleventh visit: 6 months (±31 days) post 2nd vaccination 39
Twelfth visit: 12 months (± 31 days) post 2nd vaccination 39
Laboratory Analysis 39
5. A description of the stopping rules for individuals, parts of trial and entire trial 40
6. Maintenance of trial treatment codes and procedures for breaking the codes 40
7. The identification of any data to be recorded directly in CRFs and to be considered to be source data 40

6 Selection and Withdrawal of subjects 41
1. Subjects inclusion criteria 41
2. Subjects exclusion criteria 42
3. Subjects withdrawal criteria 42
When and how to withdraw subjects from the trial 42
The type and timing of data to be collected for withdrawn subjects 43
Whether and how subjects are to be replaced 43
The follow-up for subjects withdrawn from treatment 43

7. Treatment of Subjects 43
1. Treatment to be administered including name of all products, the doses, the dosing schedule, the route of administration and the treatment periods including follow-up periods for the subjects for each product treatment group 43
2. The methods and timing of the screening tests 44
3. Medication permitted (including rescue medication) and not permitted before and /or during trial 46

4. **Procedures for monitoring subject compliance** 46
8 Assessment of Efficacy 46
1. **Specification of efficacy parameters** 46
2. **Methods and timing for assessing recording and analyzing efficacy parameters** 47
Deviation from protocol 48
Protocol amendments 48
9 Assessment of Safety 49
1. **Averse events (AE)** 49
Definitions 49
Surveillance, reporting, and documentation of adverse events 50
Documentation of solicited adverse events 50
Documentation of adverse events 51
Reporting of serious adverse events 52
Causality of adverse events 53
Severity of adverse events 54
Follow-up of ongoing adverse events and assessment of outcome 54
Follow-up of non-serious adverse events 54
Follow-up of serious adverse events 55
Treatment of adverse events 55
2. **Handling of pregnancy cases** 55
10. Statistics 56
1. **A description of the statistical methods to be employed** 56
2. **The number of subjects planned to be enrolled and reason for choice of sample size** 56
Deviation from protocol and procedures for accounting for missing data 56
11. Direct access to source data/documentation 57
12 Quality Control and Quality Assurance 57
13. Ethics 58
Good Clinical Practice 58
Regulatory authority approval 58
Ethics committee approval 58
Biobank and Data law 59
Patient informed consent 59
Removal/withdrawal of subjects from treatment or assessment 59
Notification of primary care physician 59
14 Data handling and record keeping 60
Storage and retention of study documentation 60
15 Financing and Insurance 60
16 Publication Policy 60
17 Supplements 61
18 References 62
Appendix 65
Norwegian Case Report Form 66
Norwegian Adverse Event Form 74

List of tables and figures

Table 1.1: Composition of final product....................11
Table 2.1 Examples of recent avian influenza viruses transmitted to humans....................16
Table 2.2 Overview of clinical studies with ISCOM™-adjuvanted vaccines....................19
Table 5.1 Summary of the time schedule of the trial....................33
Figure 5.2. Flow chart of the trial....................34
Table 9.1 The timing of the screening tests....................45
Table 9.2 Normal reference ranges for the screening tests....................45

List of abbreviations

AE	Adverse Event
ALAT	alanine amino transferase
ANA	anti-nuclear antibodies
ASAT	Aspartate amino transferase
ASC	antibody-secreting cells
BSL	Biological Safety Level
CHMP	Committee for Medicinal Products for Human Use
CRF	case report form
CRP	C reactive protein
EDTA	ethylenediaminetetraacetic acid
ELISA	enzyme-linked immunosorbent assay
ELISPOT	enzyme linked immunospot assay
EU	European Union
FACS	Fluorescence activated cell sorting
GCP	Good Clinical Practice
GMP	Good Manufacturing Practice
HA	haemagglutinin
HPA	Health Protection Agency
HI	haemagglutination inhibition
ICH	International Conference on Harmonisation
IM	intramuscular
IMP	Investigational Medicinal Product
ISI	Istituto Superiore di Sanità
MCV	mean cell volume
NA	neuraminidase
NIBRG-14	Depathogenised A/Vienam/1194/2004 (H5N1)
NIBSC	National Institute for Biological Standards and Control
NSD	Norsk samfunnsvitenskapelig datatjeneste
PR8	A/Puerto Rico/8/34 (H1N1) (PR8)
SAE	Serious Adverse Event
SUSAR	Suspected Unexpected Serious Adverse Reaction
SOP	standard operating procedure
SRH	single radial haemolysis
UK	United Kingdom
USA	United States of America

1. General administrative information

1. Name and address of sponsor

University of Bergen

Dr. Ph.D. Rebecca Jane Cox
Head Influenza Centre
Influenza Centre
Section for Microbiology and Immunology,
The Gade Institute,
Armauer Hansen Building,
N-5021 Bergen,
Norway

2. Name and title of person authorized to sign protocol

Dr. Ph.D. Rebecca Jane Cox

3. Name, title, address and telephone number of sponsors medical expert

Professor, dr. med. Haakon Sjursen
Consultant Infectious Diseases Unit,
Haukeland University Hospital,
N-5021 Bergen
Norway
Tel: +47 55 97 29 24
Fax: +47 55 97 29 50
E-mail: haakon.sjursen@helse-bergen.no

Dr. cand, med. Kjell Bjørn Rørvik
Consultant Infectious Diseases Unit,
Haukeland University Hospital,
N-5021 Bergen
Norway
Tel: +47 55 97 29 24
Fax: +47 55 97 29 50
E-mail: haakon.sjursen@helse-bergen.no

Professor, dr. med. Nina Langeland
Head Infectious Diseases Unit,
Haukeland University Hospital,
N-5021 Bergen,
Norway

Tel: +47 55 97 29 61
Fax: +47 55 97 29 50
E-mail: nina.langeland@helse-bergen.no

4. Name and title of investigators responsible conducting trial

Principal Investigator:

Dr., Ph.D. Rebecca Jane Cox

Clinical investigators:

Professor, dr. med. Haakon Sjursen

Dr. cand. med. Kjell Bjørn Rørvik
Professor, dr. med. Nina Langeland

5. Name, title, address and telephone number of qualified physician

Professor, dr. med. Haakon Sjursen
Consultant Infectious Diseases Unit,
Haukeland University Hospital,
N-5021 Bergen
Norway
Tel: +47 55 97 29 24
Fax: +47 55 97 29 50
E-mail: haakon.sjursen@helse-bergen.no

Professor, dr. med. Nina Langeland
Head Infectious Diseases Unit,
Haukeland University Hospital,
N-5021 Bergen,
Norway
Tel: +47 55 97 29 61
Fax: +47 55 97 29 50
E-mail: nina.langeland@helse-bergen.no

6. Name and address of collaborating laboratories

Drs. Rebecca Cox and Abdullah Madhun
Influenza Centre
University of Bergen,
The Gade Institute,
Armauer Hansen Building,

N-5021 Bergen,
Norway
Tel: +47 55 97 46 68/67
Fax: +47 55 97 46 89
E-mail: rebecca.cox@gades.uib.no, abdullah.madhun@gades.uib.no

Dr. John Wood
Division of Virology,
National Institute for Biological Standards and Control (NIBSC)
Blanche Lane,
South Mimms,
Potters Bar,
Hertfordshire
EN6 3QG,
UK.
Tel: +44 1707 641309
Fax. +44 1707 646730,
E-mail: jwood@nibsc.ac.uk

Professor Maria Zambon
Head of Respiratory Unit,
Health Protection Agency (HPA) Colindale,
Enteric, Respiratory & Neurological Virus Laboratory
61 Colindale Avenue,
London
NW9 5HT,
UK.
Tel: +44 20 8200 4400 Ext 6269,
Fax: +44 20 8205 8195
E-mail: maria.zambon@hpa.org.uk

Dr. Isabella Donatelli
Director
National Influenza Centre
Istituto Superiore di Sanità
Viale Regina Elena, 299
00161 Rome
Italy
Tel. +39-06-49903257
Fax +39-06-49902082
E-mail: isabella.donatelli@iss.it

2. Background Information

1. Name and description of investigational medicinal product

1.1 The antigen

The influenza virus strain used for the production of the drug substance is the NIBRG-14 strain, derived from A/Vietnam/1194/2004 (H5N1). The strain is a re-assortant between A/Vietnam/1194/2004 and A/Puerto Rico/8/34 (H1N1) (PR8) produced by reverse genetics at the National Institute Biological Standards and Control, (NIBSC) United Kingdom (UK). For production, the virus is grown in the allantoic cavity of embryonated hens' eggs from healthy flocks. The working virus seed lots used have been tested for the absence of bacterial, fungal, and mycoplasma contamination.

1.2 The adjuvant

To obtain the 3rd generation immune stimulating complex (ISCOM™) adjuvant, ISCOM-Matrix-A and ISCOM-Matrix-C fractions are produced from purified *Quillaja* saponin fractions A and C derived from water extracts of *Quillaja saponaria* Molina bark. ISCOM-Matrix-A and ISCOM-Matrix-C are mixed in defined proportions before addition to the antigen.

1.3 Formulation and composition

For the formation of virosomes, the influenza virus is first inactivated with beta-propiolactone and HA and phospholipids are solubilised with the detergent octaethyleneglycol onododecylether. The influenza surface antigens neuraminidase (NA) and haemagglutinin (HA) are purified and mixed with phospholipid components, namely lecithin. The NA and HA viral antigens are incorporated into the phospholipid bilayer by spontaneous formation of the virosomes induced by the step-wise removal of the detergent.

The final bulk vaccine is prepared by adding a pre-calculated amount of 3rd generation ISCOM™ to the concentrated H5N1 virosomal bulk to obtain the final vaccine composition (see Table 1.1).

Table 1.1: Composition of final product

Name of ingredients	Unit and/or formula per 0.5 mL-dose	Function	Reference to standards
Active ingredient			
Influenza virus strain: avian influenza A/Vietnam/1194/2004 NIBRG-14	1.5, 7.5, or 30 µg HA	Active ingredient	Strain provided by NIBSC, UK
Other ingredients			
NaCl	2.4 mg	Buffer substance	Ph.Eur. 3rd
Na_2HPO_4 x $2H_2O$	3.8 mg	Buffer substance	Ph.Eur. 3rd
KH_2PO_4	0.7 mg	Buffer substance	In-house specifications
3rd generation ISCOM™	50 µg	Adjuvant	In-house standard
Lecithin	3.9, 19.5, or 78.0 µg	Carrier	In-house standard
Water for injection	ad 0.5 mL	Solvent	Ph.Eur. 3rd

Interactions with other medications: No interaction studies have been conducted.

2. A summary of findings from non clinical studies that potentially have clinical significance and from clinical trials that are relevant to the trial

Non-clinical studies

Repeated dose toxicity studies with the 3rd generation ISCOM™-formulated H5N1 vaccine were performed in male and female New Zealand White albino rabbits and Sprague Dawley rats. Furthermore, safety pharmacology studies in male Sprague Dawley rats have been performed including neurobehavioral observations and automated motor activity assessment after intramuscular or intranasal administration. The data from these toxicology studies showed the absence of important toxicological findings, supporting the progression of the development to a Phase I clinical study in humans.

H5N1 influenza virus

The influenza virus strain used for the production of the drug substance is the NIBRG-14 strain,

derived from A/Vietnam/1194/2004 (H5N1). The A/Vietnam/1194/2004 strain is one of the reference viruses suitable for use as a mock-up vaccine strain [1]. Several clinical trials have been conducted with this virus and with Focetria and Prepandrix two A/Vietnam/1194/2004 containing vaccines have obtained regulatory approval by the EMEA. Both registered vaccines contain adjuvants to improve the immune response to the vaccine virus.

The ISCOM™ adjuvant

The first and second generation ISCOM™ adjuvants have been administered to almost 1000 people [2, 3] without any vaccine-related serious adverse events or clinically significant biochemical or haematological abnormalities. The most common adverse event was pain/ache at the injection site, which was mild to moderate and transient in nature (2-3 days), resolving without the need for medical intervention.

3. Summary of the known and potential risks and benefits, if any to human subjects

Pandemic H5N1 vaccines reported to the WHO at its meetings held in 2005-2008 were described as safe and well tolerated in all age groups studied

4. Description of and justification for the route of administration, dosage, dosage regimen and treatment period

Influenza vaccines are routinely administered intramuscularly (IM) or deep subcutaneously. In immunologically naïve subjects (e.g. children), two doses of vaccine are given at 4-week intervals. Influenza vaccines are standardised according to the concentration of HA, and 15µg of each strain is used in the seasonal influenza vaccines.

Sixty subjects will receive two doses of virosomal H5N1 influenza vaccine (separated by 21 ± 4 days) by intramuscular injection into the deltoid muscle. Escalating doses will be separated by a period of one week. Four groups each containing 15 subjects will receive two doses of the pandemic virosomal A/H5N1 influenza vaccine containing:

Group 1	30µg HA IM,
Group 2	1.5µg HA adjuvanted with 50µg 3rd generation ISCOM™ IM,
Group 3	7.5µg HA adjuvanted with 50µg 3rd generation ISCOM™ IM,
Group 4	30µg HA adjuvanted with 50µg 3rd generation ISCOM™ IM.

The pandemic virosomal A/H5N1 influenza vaccine (0.5 mL) is supplied as a suspension for injection in pre-filled syringes. Depending on the study group, volunteers will receive adjuvanted or non adjuvanted vaccine formulation, which will be administered intramuscularly. The intramuscular injection will be given into the centre of the deltoid muscle between the shoulder and axilla and between the back and front of the arm. Before injection, the syringe plunger will be drawn back in order to check that the injection is not administered intravascularly. After vaccination, all subjects must remain under observation by the study staff for at least 45 minutes.

5. Statement that trial will be conducted in compliance with the protocol, GCP and the applicable regulatory requirements

This study is compliant with all aspects of ICH / Good Clinical Practice and local regulations.

6. Description of population to be studied

Sixty healthy subjects aged 19-50 years will be recruited primarily from employees or students at Haukeland University Hospital or the University of Bergen. Subjects will be briefly informed of the nature of the study in a lecture and by the subject information letter. They will be enrolled during subsequent one to one interviews when they will receive a detailed explanation of the study protocol. If the subject meets the inclusion and does not meet the exclusion criteria and signs the informed consent form, they will be enrolled in the trial and allocated a unique subject identification number. Subjects will be considered eligible to enter the study provided they satisfy the following criteria:

Inclusion criteria

- Healthy volunteers (as concluded from the medical history, physical examination, and clinical judgment) who are between 19 and 50 years old
- Females using a reliable method of contraception (from 4 weeks prior to the first vaccination until 4 weeks after the second vaccination) and a negative urine pregnancy test will be required before administration of each dose of vaccine
- Signed informed consent

- Subjects able to understand and comply with the study protocol and complete the Adverse Event Form
- Subjects able to attend the scheduled visits
- Subjects with normal pre-screening values. If a subjects prescreening samples lie outside the reference values he/she will only be included in the study based upon the medical evaluation of the clinical investigator

Exclusion criteria

- Persons with a history of anaphylaxis or serious reactions to any vaccine
- Person with known hypersensitivity to any of the vaccine components
- Persons who have had a temperature >38°C during the previous 72 hours
- Persons who have had an acute respiratory infection during the last 7 days
- Women who are pregnant or breast-feeding
- Persons with chronic illness at any stage that could interfere with trial conduct or compliance
- Persons who have received blood products or immunoglobulins parenterally during the previous 3 months
- Persons who have been vaccinated with any vaccine during the 4 weeks preceding the first trial vaccination
- Persons with known or suspected immunosuppressive disease or who use systemic immunosuppressive drugs
- Persons taking immunostimulant therapy
- Persons involved in another clinical trial during the last month
- Suspected non-compliance

7. References to literature and data that are relevant to the trial and that provide background for the trial

Background

Influenza virus is a globally important respiratory pathogen, which annually causes high levels of morbidity and mortality. Three types of influenza virus have been identified (A, B, and C), but only influenza A and B cause overt disease and result in regular outbreaks or epidemics. Influenza A viruses are further subdivided into subtypes based on the surface antigens, haemagglutinin and

neuraminidase. Today, 16 subtypes of HA (H1–H16) and 9 subtypes of NA (N1–N9) have been found in influenza A viruses, and all the subtypes are found in aquatic birds.

Antigenic shift and drift

Influenza virus in man continuously undergoes antigenic changes in the surface glycoproteins (HA and NA) to escape the host's acquired immunity, so-called antigenic drift. Antigenic drift is responsible for the inter-pandemic outbreaks of influenza and consequently the vaccine has to be annually updated.

Aquatic birds are the natural reservoir for influenza A viruses, harbouring all known subtypes, and constitute a risk for the introduction of new influenza A subtypes to man. The appearance of such an influenza A virus (with novel surface glycoproteins HA and/or NA) able to infect and spread in an immunologically naïve human population is designated as antigenic shift. This occurs at infrequent and unpredictable intervals resulting in a pandemic with the potential to cause exceptionally high levels of global morbidity and mortality. There have been three pandemics in living memory (1918, 1957 and 1968) and although each was associated with worldwide spread of virus, the highest death toll was seen in 1918-19, when 20-40 million people died, many of whom were young healthy adults. Two of the antigenic shifts, which occurred in the 20th century, were probably due to reassortment between human and avian viruses possibly via an intermediate host (most likely pigs) serving as a mixing vessel.

Infection of man by avian influenza virus

It is generally accepted that the next pandemic could come from an avian influenza infecting humans, although it is not clear if the ability for inter-human spread will be acquired by mutation or reassortment. In 1997, an avian influenza H5N1 virus was transmitted from birds to 18 people in Hong Kong, who became seriously ill and one third of them died. If this highly pathogenic virus had been able to transmit from person to person, a new influenza pandemic could have occurred with potentially disastrous global effects. This pandemic warning prompted health authorities worldwide to face the threat of a possible influenza pandemic. Since this time a number of other avian influenza subtypes (H5, H7, H9 see Table 2.3) have crossed the species barrier into man by direct transmission from birds without the need for an intermediate mixing vessel resulting in illness and in some cases high mortality. In 2003, the avian influenza H5N1 virus re-emerged and has caused large severe outbreaks in poultry mainly in the Far East. Since

January 2004, 387 human cases of H5N1 infection have been reported in 15 countries with Indonesia reporting 137 of confirmed cases of these 112 have been fatal. The ongoing threat posed by avian influenza highlights the need for global preparedness.

Table 2.1 Examples of recent avian influenza viruses transmitted to humans

Year	HA Subtype	Country/ Region	No. of cases (No. of deaths)	Severity of illness	Comments
1997	H5N1	Hong Kong	18 (6)	serious	Highly pathogenic H5N1 in poultry markets. Virus transmitted directly to humans from infected poultry. All poultry destroyed.
1999	H9N2	Hong Kong<br>China	2 children<br>5	mild<br>mild	
2003	H7N3	Italy	7	asymptomatic	Occupational exposure of poultry workers
2003	H5N1<br><br>H7N7	Vietnam<br>China<br>Netherlands<br>Belgium	3 (3)<br>1(1)<br>83 (1)	serious<br><br>mild	 <br>Highly pathogenic H7N7 in chickens. Affected birds slaughtered.
2004<br>2005<br>2006<br>2007<br>2008	H5N1	2 countries<br>5 countries<br>9 countries<br>9 countries<br>5 countries	46 (32)<br>98 (43)<br>115 (79)<br>88 (59)<br>36 (28)	serious	Highly pathogenic H5N1 outbreaks.<br>Human infection due to exposure to infected birds.

Influenza subtypes H5 and H9 have been generally associated with respiratory disease, whereas H7 has been associated with conjunctivitis. Adapted from [4; 5].

Influenza Vaccines

While antivirals play a role in disease control, vaccination remains the most effective prophylactic measure to prevent morbidity and mortality (reviewed by [6]). Two types of influenza vaccine are available; inactivated vaccine delivered deep subcutaneously or intramuscularly and live attenuated vaccine administered intranasally. Inactivated influenza vaccines have been licensed for many decades and are 60-80% effective in preventing disease with homologous or closely related strains. In some cases, vaccination may not prevent influenza illness, however it reduces the severity and duration of the illness. Inactivated influenza vaccines are used routinely every

year and they are considered to be safe. Inactivated vaccines are available in whole, split (chemically disrupted), and subunit (purified surface glycoproteins) formulations.

Current inactivated vaccines are produced by propagation in embryonated hens' eggs. The allantoic fluid is harvested, and the virus is concentrated and highly purified, then inactivated with formaldehyde or beta-propiolactone. The availability of embryonated hens' eggs is a limiting factor in vaccine production and the global manufacturing capability is not expected to meet pandemic vaccine requirements, thus it is important to develop dose sparing strategies by using effective adjuvants. The use of reverse genetics technology can save a considerable amount of time in production of a pandemic vaccine. Traditionally, influenza vaccines have been made by choosing a virus isolate which closely matches circulating strains, and introducing its surface glycoprotein genes (HA and NA) into the genetic background of the PR8 virus. Seed viruses are produced by co-infection of the two "parent" viruses, and screening for the progeny of interest. This screening from a possible 256 progeny genotypes is time consuming, and may be unsuccessful. The use of reverse genetics allows viruses to be constructed with a prescribed combination of genes, following combination of plasmid DNAs encoding separate RNA segments. Genetic manipulation allows engineering of the DNAs to create viruses altered in specific genes for example attenuation of highly pathogenic avian viruses to create a depathogenised virus, which can be used as a vaccine strain (reviewed in [7]. A rapid response to a pandemic threat by production of a safe, effective vaccine is one of the most important objectives in pandemic control. The potential to save valuable time in preparing a pandemic influenza vaccine and provide essential information on the optimal use of the vaccine could have an important impact on the public health. Should a pandemic occur today, a safe, immunogenic and appropriately formulated vaccine is urgently needed which could be rapidly prepared in sufficient quantities. Current seasonal influenza vaccines contain 15 µg HA from each of the three seasonal strains. In contrast, very high antigen doses (up to 90 µg HA) are required for non-adjuvanted split virus or recombinant pandemic H5 vaccines to elicit an antibody response sufficient to meet the CHMP criteria [8, 9]. This underlines the need for effective adjuvants to enhance the immune response to split or subunit pandemic influenza vaccines. The best candidate vaccine formulation currently available is an adjuvanted inactivated virus vaccine (reviewed in [10; 11; 12]).

Virosomal vaccine

Virosomal influenza vaccines are virus-like particles, consisting of reconstituted influenza virus envelopes, lacking the genetic material of the native virus. Crucell Berna Biotech Ltd. produces a virosomal influenza vaccine (Inflexal® V) which is recommended for all age groups (above 6 months old). Inflexal® V was originally introduced in 1997, and is registered in 35 countries and more than 41 million doses have been administered confirming its safety profile, in 11 influenza seasons.

ISCOM™ adjuvant

ISCOM™ technology is a structure for antigen presentation with immunomodulatory properties. The complex is formed by strong cholesterol binding to *Quillaja* saponins (*Quillaja saponaria Molina*) forming 12 nm rings. These rings are held together by lipid based (e.g. phosphatidyl choline) hydrophobic interactions, to form spherical particles 40 nm in diameter. ISCOM™-like adjuvants have been developed over many years from the first generation ISCOM™ into a second and third generation of ISCOM™ adjuvants. The first and second generation of ISCOM™ adjuvants have been extensively tested in human clinical trials without the occurrence of major adverse effects. Pre-clinical studies in in *vitro* systems and toxicology studies in animals have demonstrated an improved safety profile for the third generation ISCOM™ adjuvants [13].

In the first generation ISCOM™ preparations the antigen(s) were incorporated in the matrix. In the second generation ISCOM™ preparations (ISCOMATRIX™ or MATRIX) the MATRIX structure is pre-formed and the antigen is added later and is thus not incorporated into the structure. In the third generation ISCOM™ preparation Fraction A and Fraction C of purified *Quillaja* saponins are incorporated separately in two distinct matrices called MATRIX-A and MATRIX-C, respectively. The two MATRIX A and C adjuvants are mixed in a pre-set proportion of 10A + 1C to yield the third generation ISCOM™, called ISCOM™-M. Most saponin based adjuvants tested in humans have been based on QS21 (Antigenics) or MATRIX-C (ISCOMATRIX™, CSL Ltd). The Fraction-C and QS21 preparations are essentially identical. They are both potent adjuvants particularly in the form of ISCOM™s. The ISCOM™ formulation abolishes or substantially reduces the inherent haemolytic activity of the saponin. The Fraction-A (or the corresponding QS7) is a non-toxic saponin with low or no haemolytic activity and with a comparatively low adjuvant activity. However by the addition of MATRIX-A particles to the MATRIX-C formulation it became possible to reduce the amount of the more reactive component

(MATRIX-C) to less than 10%. Mice are very sensitive to saponins. With the third generation ISCOM™ formulation, mice have no side reactions at doses 5-10 times higher than the lethal dose, if used according to the second generation formulation. A subcutaneous dose of 100 µg (twice the proposed human dose) is well tolerated. A suitable dose with potent adjuvant effect is 5-10 µg in the mouse model. Other sensitive animals like cats have been dosed with 30-60 µg MATRIX-M and dogs dosed with 75-150 µg without adverse reactions. A brief overview of the human clinical trials with ISCOM™-adjuvanted vaccines is shown in Table 2.4.

Table 2.2 Overview of clinical studies with ISCOM™-adjuvanted vaccines

**Antigen**	**No. of subjects receiving ISCOM™ adjuvant**	**Route**	**No. of immunisations**	**Dose of ISCOM™ adjuvant (µg)**	**Dose of antigen (µg)**	**References**
Influenza	44	IM	1	50, 75	45	[14]
Influenza	17	IM	1	50	45	[15]
NY-ESO-1	22	IM	3	12, 36, 120	10, 30, 100	[16]
HPV16 E6E7	24	IM	3	120	20, 60, 200	[17])
HPV16 E6E7	36	IM	3	60, 120	5, 25, 70, 240	[17]
HCV Core	24	IM	3	120	5, 20, 50	[3]
Influenza	55	IM	1	60	15, 45	[3]
Influenza	24	IM	2	100, 500, 1000	90	[3]

Safety of inactivated influenza vaccine

Millions of doses of inactivated egg-grown influenza vaccine are administered each year, and the vaccine has excellent safety and tolerance profiles [18; 19], with very low numbers of adverse reactions reported. Mild local reactions consisting of tenderness and redness at the injection site are often observed after inactivated influenza vaccination in more than 50% of healthy adults, although these are mild and rarely interferes with normal activities [20]. Although systemic reactions such as fever, malaise, myalgia and headache are recorded, controlled trials have not been able to establish a difference between systemic symptoms associated with receipt of vaccine and those associated with receipt of placebo [20]. Immediate (allergic) reactions or hypersensitivity to influenza vaccines occur extremely rarely (in Norway, the incidence of anaphylactic shock for all types of vaccine is estimated to be less than 5 cases per 800,000 [21]. There have been reports of an association between Guillain Barré Syndrome and influenza vaccine, but it is estimated that the overall risk is only 1 case per million persons immunised

[22]). The inactivated influenza vaccine for this trial is a virosomal influenza vaccine produced, standardised and safety tested by Crucell Berna Biotech Ltd. using conventional procedures, so the above safety considerations will also apply.

Kinetics of the immune response to inactivated influenza vaccine

Antibodies to the surface glycoproteins, HA and NA, of seasonal influenza strains are associated with resistance to infection, whereas antibodies to the conserved internal antigens, M and NP, are not protective [23]. The cytotoxic T-cell response is mainly directed against the M and NP proteins and is important for clearance of the virus and recovery from illness.

Vaccination with inactivated vaccine results in both local and systemic responses. The serum antibody response increases as early as 2-6 days after influenza vaccination in primed subjects [24] and peaks at approximately 2 weeks after vaccination when 90% of vaccinees have protective antibody titres [24; 20]. The serum antibody response then wanes over time and is generally two-fold lower 6 months after vaccination [25]. A rapid increase in local secretory IgA antibody in the oral fluid is also observed after parenteral inactivated influenza vaccination but this antibody response only remains elevated for 3 weeks after vaccination [26, 27]. Influenza-specific antibody-secreting cells (ASC) appeared earlier, at approximately 7 days post-vaccination, in the blood than serum antibody response [24] and consisted predominantly of IgG and IgA. In young children (2 to 3 years old), previous natural priming by influenza infection was essential to mount strong antibody and antibody secreting cell responses in the peripheral blood [28, 29].

European Union (EU) requirements for influenza vaccines

The Committee for Medicinal Products for Human Use (CHMP) has defined a set of regulatory criteria influenza vaccines need to meet in order to obtain a marketing license in the European Economic Area [30]. The vaccine efficacy is tested by the haemagglutination inhibition (HI) [31], or single radial haemolysis (SRH) assays [32]. An HI titre ≥ 40 (or an equivalent SRH > $25mm^2$) indicates 50% protective levels of serum antibody against influenza infection in man. The EU requirements for harmonisation of influenza vaccines require annual clinical trials for licensing. The vaccine should fulfil one of the following evaluation criteria for each strain in adults aged 18-60:

1. The number of seroconversions or significant increases in anti-haemagglutinin antibody titre should be > 40%;

2. Mean geometric increase >2.5;
3. The proportion of subjects achieving an HI titre ≥40 or SRH > 25mm^2 should be >70%.

There are no requirements for inactivated vaccines to evoke a cellular immune response. The World Health Organisation (WHO) has prepared a number of recommendations for increased preparedness and research [33]. The CHMP require pandemic vaccine to meet all the current evaluation criteria for marketing authorisation. They also recommend measuring neutralising antibodies, and suggest more detailed analysis of the immune response for example detection of antibodies directed against neuraminidase and cell mediated responses [34].

Pandemic influenza vaccines

Parenterally administered inactivated influenza vaccines have been used for many decades and extensive information is available on their quality, safety and efficacy. These vaccines elicit an effective systemic immune response in individuals previously primed by natural infection but much less information is available on the immune response to influenza vaccines containing novel subtypes. During an influenza pandemic, vaccines will be a key intervention step in protecting the population. The type of vaccine, the amount of antigen, the route of administration, the number of doses and the incorporation of an adjuvant, in addition to the immunological status of the recipient, will all influence the immune response.

The use of a single dose regimen with a novel subtype is unlikely to be suitable in an immunologically naïve population, whereas a two dose regime more often results in seroconversion and fulfilment of the CHMP requirements [35-38]. The current influenza vaccine manufacturing capacity will not be sufficient to meet the global demand for a pandemic vaccine, particularly if two doses of vaccine are required to elicit an adequate antibody response [8, 38-42]. Whilst whole virus vaccines have proved more immunogenic in unprimed adults than split or subunit vaccines [38, 43], most vaccine manufacturers are technically unable to convert their current split or subunit vaccine production process to whole virus production. Current seasonal influenza vaccines contain 15 µg HA from each of the three seasonal strains. In contrast, very high antigen doses (up to 90 µg HA) are required for non-adjuvanted split virus or recombinant pandemic H5 vaccines to elicit an antibody response sufficient to meet the CHMP criteria [8, 9]. Aluminium-salt adjuvants are readily available and inexpensive, and have been shown to modestly augment the antibody response after two doses of candidate pandemic influenza vaccines, either split [42] or whole virus formulations [35, 43]. However, other trials found that

aluminium adjuvant did not significantly enhance the antibody response to candidate H5 vaccines [41, 44, 45]. The most promising adjuvants are the proprietary oil-in-water emulsion systems such as MF59, AS and AF03, which greatly enhanced homologous and cross reactive antibody responses after H5 vaccination [36, 37, 40, 45, 46] even at lower antigen concentrations (3.8 - 7.5 μg HA). Preclinical studies of virosomal H5N1 vaccine adjuvanted with 3rd generation ISCOM™ have shown equal or greater ability to dose spare to the oil in water emulsion systems [13].

There are more than 70 clinical trials of candidate pandemic influenza vaccines, which have been completed or are ongoing [47]. To date pandemic influenza vaccines have been reported as safe and well tolerated, with most trials performed in healthy adults. A number of pandemic vaccines have met regulatory approval in Europe, the USA and Australasia.

Importantly, the goal of pandemic vaccination is to elicit appropriate immunological effector mechanisms to reduce viral replication and thus viral shedding and provide protection against serious illness and death upon infection by pandemic influenza. Protective efficacy studies using pandemic candidate H5 vaccines in animal models have shown that correlates of resistance to serious illness and death to avian viruses may not be solely reflected by levels of circulating serum antibodies [48-51]. These findings raise concern about the use of the CHMP criteria for assessing surrogate correlates of protection to candidate pandemic vaccines and there is therefore a need for more research to allow a better understanding of the immune response after pandemic influenza vaccination in man.

PANFLUVAC consortium

The WHO commissioned H5N1 seed virus NIBRG-14 virus, which is derived from the genetically modified A/Vietnam/1194/2004 (H5N1) virus will be used in this study. Plasmids for the H5 HA and N1 NA were cloned from A/Vietnam/1194/2004 (H5N1). These were used with the six PR8 backbone plasmids and four PR8 polymerase expression plasmids to generate a candidate H5N1 vaccine reference strain using the reverse genetics procedures. The cloned H5 HA was engineered to remove the polybasic amino acids at the cleavage site (a molecular correlate of pathogenicity in birds and mice). This rescued virus - NIBRG-14 - was tested for lack of pathogenicity in chickens and ferrets, before removal from the Biological Safety Level (BSL) 4 containment and being assigned a BSL2+ status.

Berna Biotech Ltd. has produced an H5N1 virosomal influenza vaccine. The NIBRG-14 virus was propagated in the allantoic cavity of embryonated hens' eggs. The virus was inactivated by beta-propiolactone and HA and phospholipids were solubilised with the detergent octaethyleneglycol

onododecylether. The influenza surface antigens NA and HA were purified and mixed with lecithin, and incorporated into virosomes by the step-wise removal of the detergent. The vaccine will be formulated with or without the 3rd generation ISCOM™ adjuvant for parenteral administration. The vaccine will be administered as twice the normal human dose (30μg HA) with and without adjuvant, and in a dose sparing manor of half (7.5μg HA) and one tenth (1.5μg HA) of the normal human dose with adjuvant

There are five key criteria, which need to be addressed in clinical trials of pandemic influenza vaccines in man [52]; 1) safety, 2) immunogenicity, 3) kinetics of the response, 4) long lasting immunity and 5) cross reactive immunity to influenza H5 strains. Furthermore, there is a clear need for improved understanding of the immune response elicited in human volunteers after vaccination with candidate pandemic vaccines including the quality and repertoire of the antibody response. These five criteria will be addressed in this trial as follows: The safety and tolerability of the H5N1 vaccine will be investigated locally and systemically and by using haematological, biochemical and immunological screening tests. The immunogenicity of the H5N1 vaccine will be assessed through the induction of local and systemic antibody and cellular immune responses. In a pandemic situation, an important aspect is the rapidity of the immune response to the H5N1 vaccine so the detailed kinetics of the immune response will be investigated. The capacity of the vaccine to elicit long lasting immunity and cross reactive immunity to H5 viruses will also be evaluated. Furthermore, the quality of the immune response induced by the vaccine will be studied.

4. TRIAL OBJECTIVES AND PURPOSE

The objectives and purpose of this trial are:

1) Evaluation of the local and systemic safety and tolerability of parenterally administered virosomal H5N1 influenza vaccine, with and without 3rd generation ISCOM™ adjuvant.
2) To evaluate the immunogenicity of a virosomal H5N1 influenza vaccine including:

- Examine the effect of the use of adjuvant and different antigen strengths to provide a dose sparing approach
- Provide a detailed time course of the immune response to vaccination with a novel influenza subtype including the kinetics of the T-cell, B-cell and antibody responses

- Investigate the long lasting immunity induced by the vaccine
- Examine the ability of the vaccine to induce cross-reactive immunity to H5 strains of different clades
- Evaluate the quality of the immune response induced by the vaccine.

5 TRIAL DESIGN

1. A specific statement of the primary endpoints and secondary endpoints to be measured by the trial

The primary endpoints of the trial are the local and systemic adverse events and tolerability of parenterally administered virosomal H5N1 influenza vaccine with or without 3rd generation ISCOM™ adjuvant. The secondary endpoints are the evaluation of the immunogenicity of a non-adjuvanted and 3rd generation ISCOM™ adjuvanted virosomal H5N1 influenza vaccine and the ability of the vaccine to meet the CHMP evaluation criteria.
The immunogenicity of the H5N1 vaccine will be assessed through the induction of specific local and systemic antibody and cellular immune responses, and analyses of the epitopes to which the response is directed. Furthermore the capacity of the vaccine to elicit cross reactive and long lasting immunity will be evaluated. In a pandemic situation, an important aspect is the rapidity of the immune response to the H5N1 vaccine and therefore the detailed kinetics of the immune response will be investigated.

A description of the type/design of trial to be conducted

The clinical trial will be an open phase I dose escalating pilot study in 60 healthy subjects aged 19 -50 years who will receive two doses of vaccine, separated by 21 ± 4 days, by intramuscular injection into the deltoid muscle. Escalating doses will be separated by a period of one week. Four groups of 15 subjects will receive two doses of pandemic virosomal A/H5N1 influenza vaccine containing

Group 1	30µg HA IM,
Group 2	1.5µg HA adjuvanted with 50µg 3rd generation ISCOM™ IM,
Group 3	7.5µg HA adjuvanted with 50µg 3rd generation ISCOM™ IM,
Group 4	30µg HA adjuvanted with 50µg 3rd generation ISCOM™ IM.

The second vaccine dose will contain the same quantity of antigen and adjuvant as in the first dose. Blood and oral fluid samples will be collected at days 0, 3, 7, 14 and 21 after 1st and 2nd doses of vaccine. This study is an open study, however the trial samples will be coded so that only the investigators are aware of the code. Serological assays will be conducted blinded by the reference laboratories, National Institute for Biological Standards and Control (NIBSC), Health Protection Agency (HPA) and Istituto Superiore di Sanità (ISI). The code will be broken when all immunological assays have been performed at the reference laboratories (NIBSC, HPA, and ISI) on the samples collected up to 21 days after second dose of vaccine. Blood samples will also be collected at 6 and 12 months after vaccination. The kinetics of the immune response will be studied at the University of Bergen.

2. A description of measure taken to avoid/minimize bias

The study will be an open phase I dose escalating pilot study and the subjects will be randomly assigned to one of 4 vaccination groups, containing a similar sex and age distribution. All trial blood samples will be coded and serological assays to be conducted at NIBSC, HPA and ISI will be performed blinded.

3. A description of the trial treatment and dosage and dosage regimen of the investigational medicinal product.

Subjects will be immunised twice by intramuscular injection into the deltoid muscle, separated by 21 ± 4 days. Escalating doses will be separated by a period of one week. Four groups of 15 subjects will receive two doses of the pandemic virosomal A/H5N1 influenza vaccine containing:

Group 1	30µg HA IM
Group 2	1.5µg HA adjuvanted with 50µg 3rd generation ISCOM™ IM
Group 3	7.5µg HA adjuvanted with 50µg 3rd generation ISCOM™ IM
Group 4	30µg HA adjuvanted with 50µg 3rd generation ISCOM™ IM

Each vaccine dose will be initially administered to 5 subjects in each group (Figure 5,1). If no serious adverse or important medical events are reported in the week after immunisation the remaining 10 subjects in each group will be vaccinated. If a suspected unexpected serious adverse

reaction (SUSAR) occurs then vaccination will be suspended to allow further evaluation (see section 9 Safety Assessments).

Figure 5.1. The timeline for the trial.

Group	n=	Trial week 1	2	3	4	5	6	7	8	9	10	11	12
1	5	Pre-screen		1st dose			2nd dose						
	10		Pre-screen		1st dose			2nd dose					
2	5		Pre-screen		1st dose			2nd dose					
	10			Pre-screen		1st dose			2nd dose				
3	5			Pre-screen		1st dose			2nd dose				
	10				Pre-screen			1st dose		2nd dose			
4	5				Pre- screen		1st dose			2nd dose			
	10					Pre-screen			1st dose		2nd dose		

Group 1 = 30 μg HA
Group 2 = 1.5μg HA adjuvanted with 50μg 3rd generation ISCOM™
Group 3 = 7.5μg HA adjuvanted with 50μg 3rd generation ISCOM™
Group 4 = 30μg HA adjuvanted with 50μg 3rd generation ISCOM™

During the trial up to 12 blood (by venipuncture) and 11 oral fluid samples will be collected at intervals after vaccination. Blood samples will be collected for both screening and trial purposes. Screening samples will be used to assess the safety of the vaccine by monitoring basic haematological, biochemical and immunological parameters. Blood samples will be collected prior to vaccination (prescreen), and at 3 and 21 days after 1st and 2nd dose of vaccine to examine for acute and longer-term adverse reactions. The screening test results will be stored in NetLab database and a hard copy will be kept in the patient information folder.

The trial blood and oral fluid wash samples will be used to evaluate the antibody, T and B cellular responses induced after vaccination. The oral fluid sample will be collected using an absorbent pad placed on the gum to absorb the oral fluid for two minutes (Orasure™). Blood and oral fluid samples will be collected at days 0, 3, 7, 14 and 21 after 1st and 2nd doses of vaccine to allow investigation of influenza specific cellular and serum antibody responses, as previously described [24, 26, 28, 53-55]. These samples will be allocated a unique subject identification number, and only information on the age; sex and previous influenza vaccination history of each subject will

be available to the scientific research team. Serum samples will be sent to the three reference laboratories in Europe for studying the antibody responses.

Sera will be separated from clotted blood samples and plasma will be separated from CPT herparinised blood, aliquoted and stored at -80 °C or -20°C for use in the HI, SRH, neutralisation assay and enzyme-linked immunosorbent assay (ELISA) and other assays deemed appropriate. Lymphocytes will be separated from CPT herparinised blood samples and used directly in the relevant immunological assays e.g. enzyme linked immunospot assay (ELISPOT) and for evaluation of the B and T cellular responses by e.g. flow cytometry (FACS) and cytokine production by multiplex ELISA and FACS. Excess lymphocytes will also be stored in liquid nitrogen for use in relevant immunological assays e.g. *in vitro* activation assays at the University of Bergen.

Study treatments

Test investigational medicinal products (IMPs)

The investigational medicinal products (IMPs) have been developed and manufactured by the Crucell Holland BV Group Company Berna Biotech Ltd. The IMPs have been produced under aseptic conditions and according to Good Manufacturing Practice (GMP). The IMP lots used in this study are tested and released by the quality control department of Berna Biotech Ltd. All test results have been validated by the quality control department of Berna Biotech Ltd.

The quality control standards and requirements for the IMPs are described in separate release protocols/Certificate of Analysis and the required approvals have been obtained.

The test IMPs are formulated as virosomes containing HA antigen doses of 1.5, 7.5, and 30.0 µg of the NIBRG-14 strain, respectively, and 50 µg adjuvant (third generation ISCOM™). In addition, IMP formulated as virosomes and containing a HA antigen dose of 30.0 µg of the NIBRG-14 strain without adjuvant are provided by Crucell Berna Biotech Ltd. All IMPs are formulated as watery suspension and each dose is diluted in a volume of 0.5 mL.

All IMPs are presented as suspension in a pre-filled syringe (type I glass) with a needle size of 25G and 5/8" or 0.5mm x 16 mm for intramuscular administration. Vaccination in Groups 1-4 will be in an open fashion, based on a randomisation list prepared by the sponsor or her nominee.

**Name**	IF 196	
**Composition**	H5N1 influenza vaccine (per 0.5 mL dose):	
**Active ingredients**	Haemagglutinin antigen of avian influenza A/Vietnam/1194/2004 NIBRG-14	30.0 µg
**Excipients**	Lecithin	78.0 µg
	Sodium chloride	2.4 mg
	Di-sodium-hydrogen-phosphate-dihydrate	3.8 mg
	Potassium di-hydrogen-phosphate	0.7 mg
	Water for injection	ad 0.5 mL
**Dose and frequency**	Two doses on Day 1 and Day 21±4	
**Route of administration**	IM (M. deltoideus)	

**Name**	IF 197	
**Composition**	ISCOM™-adjuvanted H5N1 influenza vaccine (per 0.5 mL dose):	
**Active ingredients**	Haemagglutinin antigen of avian influenza A/Vietnam/1194/2004 NIBRG-14	1.5 µg
	Third generation ISCOM™	50.0 µg
**Adjuvant Excipients**	Lecithin	3.9 µg
	Sodium chloride	2.4 mg
	Di-sodium-hydrogen-phosphate-dihydrate	3.8 mg
	Potassium di-hydrogen-phosphate	0.7 mg
	Water for injection	ad 0.5 mL
**Dose and frequency**	Two doses on Day 1 and Day 21±4	
**Route of administration**	IM (M. deltoideus)	

**Name**	IF 198	
**Composition**	ISCOM™-adjuvanted H5N1 influenza vaccine (per 0.5 mL dose):	
**Active ingredients**	Haemagglutinin antigen of avian influenza A/Vietnam/1194/2004 NIBRG-14	7.5 µg
**Adjuvant**	Third generation ISCOM™	50.0 µg
**Excipients**	Lecithin	19.5 µg
	Sodium chloride	2.4 mg
	Di-sodium-hydrogen-phosphate-dihydrate	3.8 mg
	Potassium di-hydrogen-phosphate	0.7 mg
	Water for injection	ad 0.5 mL
**Dose and frequency**	Two doses on Day 1 and Day 21±4	
**Route of administration**	IM (M. deltoideus)	

**Name**	IF 199	
**Composition**	ISCOM™-adjuvanted H5N1 influenza vaccine (per 0.5 mL dose):	
**Active ingredients**	Haemagglutinin antigen of avian influenza A/Vietnam/1194/2004 NIBRG-14	30.0 µg
**Adjuvant**	Third generation ISCOM™	50.0 µg
**Excipients**	Lecithin	78.0 µg
	Sodium chloride	2.4 mg
	Di-sodium-hydrogen-phosphate-dihydrate	3.8 mg
	Potassium di-hydrogen-phosphate	0.7 mg
	Water for injection	ad 0.5 mL
**Dose and frequency**	Two doses on Day 1 and Day 21±4	
**Route of administration**	IM (M. deltoideus)	

IMP accountability

The pharmacist will confirm receipt of the clinical supplies by signing the acknowledgement of receipt form provided. At any time the numbers of supplied, used, and remaining doses have to match. It must be possible to reconcile delivery records with those of used and unused stocks. Any discrepancies must be accounted for. It is not permitted to use supplies for purposes other than those specified in this Study Protocol.

The vaccine will be stored between +2 and +8°C in the Hospital Pharmacy, which is a safe and locked area with no access to unauthorised personnel. The pharmacist will dispense the vaccine for each day's vaccination to the clinical investigator or his nominee. During the vaccination session a sufficient number of vaccine doses for the whole day will be kept in a temperature-monitored small refrigerator, which is located at the vaccination clinic. At the end of the vaccination session (usually in the evening) the unused vaccine doses are returned to the pharmacy (central cool storage room). The pharmacist will maintain accurate records of IMP dispensed to the clinical investigator. The clinical investigator will maintain accurate record of product inventory at the site, dispensed IMP recording date and time of vaccine administered, vaccine name, doses, to whom it is dispensed (subject by subject accounting), and accounts of any product accidentally destroyed. The pharmacist will retain all unused or expired product until the accountability data has been confirmed. At the conclusion of the study, all unused IMP supplies will be returned to Crucell Berna Biotech Ltd. An overall summary of all IMP supplies received, used, and returned will be prepared at conclusion of the study.

Preparation and Administration

The vaccine will be administered by the clinical investigator or his nominee, or the study nurses. Full compliance as per protocol should thus be obtained.
Before use, the vaccine will be removed from the refrigerator and placed at room temperature for 5 and 10 minutes. Prior to injection, it will be gently shaken. The vaccine will be injected intramuscularly into the deltoid region (on the opposite arm to that of the blood sampling). To ensure intramuscular injection, the subject will be asked to relax and the needle will be inserted at the center of the deltoid muscle between the shoulder and axilla and between the back and front of the arm. Before injection, the syringe plunger will be drawn back in order to check that the injection is not administered intravascularly. The site and side of injection will be recorded in the patient information and in the case report form (CRF).

Precautions for Use

Vaccination must not be performed in subjects allergic to any IMP component.

Logistics

Labeling and Packaging

Packaging and labeling will be conducted according to Good Manufacturing Practice (GMP).
The test IMPs will be packed in labeled boxes. The outer container will contain the following information: study number, IMP or code name, potency/strength, route of administration, storage, lot number, expiry date, instructions for administration, "For clinical trial use only". The GMP guideline Volume 4, Annex 13 for Investigational Products and the local law will be followed. We will apply for the use of English labeling as the study drugs will be handled only by healthcare professionals

Shipment Conditions

The clinical investigator will be personally responsible for product management or will designate a person who will be responsible for product management.
The principle or clinical investigator will determine the dates and times of delivery of products and forward the information to Clinical Operations at Crucell Berna Biotech Ltd. IMPs will then be shipped to the Hospital Pharmacy, Haukeland University Hospital by courier according to the predetermined schedule.
The person in charge of the shipment of IMP at Crucell Berna Biotech AG will issue a dispatch note with acknowledgement of receipt as part of the shipment. On delivery of the products at the Hospital Pharmacy, the pharmacist or delegated person in charge of product receipt will check whether the IMP arrived in good order. The acknowledgement of receipt will be signed and dated by the person in charge of product management and will be faxed to Crucell Berna Biotech Ltd. In addition, if the temperature loggers show that the cold chain has been broken during transport ,the temperature loggers sent along with the IMP will be returned immediately according to instructions given in the shipment for read-out to Crucell Berna Biotech Ltd. No vaccinations will be performed before confirmation of maintaince of the cold chain from the manufacturer Crucell Berna Biotech Ltd.

Storage Conditions

The test IMPs must be stored in a safe and secure area at the Hospital Pharmacy with no access for unauthorised personnel. It must be kept refrigerated (+2°C to +8°C) and must not be frozen. Storage temperature should be monitored daily and documented on the appropriate form during

the entire duration of the trial. A back-up refrigerator is available in case of failure of the main refrigerator.
During the vaccination session a sufficient number of vaccine doses to be used for the whole day will be kept in a temperature-monitored small refrigerator, which is located at the vaccination clinic. The temperature must be recorded on a vaccine temperature log daily on the vaccination days.
In case of temperature <+2°C or >+8°C, Crucell Berna Biotech AG has to be notified immediately. No vaccines stored outside the foreseen storage conditions of +2 to +8°C has to be used prior to Crucell Berna Biotech's explicit approval. In addition, the investigator or the responsible person should inform the principle investigator.

Replacement Doses

In addition to the doses for the planned number of subjects, extra doses (approximately 10%) will be supplied to replace unusable ones. In case of broken or unusable containers, the investigator should use a replacement dose. The use of any replacement dose and the reason for using it must be recorded on the accountability form.

Return of Unused Products

Unused products will be returned to Crucell Berna Biotech AG at the end of the vaccination period together with a copy of the accountability form and the "Return of unused products" form. Empty boxes of the IMP will be destroyed on site, once they have been accounted.

4. The expected duration of subject participation and a description of the sequence and duration of all trial periods.

Each subject will be involved in the trial for a period of 12-14 moths. The main part of the trial will last 10 weeks, but the subjects will be asked to give trial blood samples at 6 and 12 months after first dose of vaccine to examine the longevity of the immune response.

A summary of the proposed study plan is given below in the Table 5.1 and Figure 5.2.

Table 5.1 Summary of the time schedule of the trial

	Pre-screening Day -14	Vaccination Day 0	Day 3	Day 7	Day 14	Vaccination Day 21	Day 24	Day 28	Day 35	Day 42	6 months	12 months
**Allowable time variation (±days)**	**±12**		**±1**	**±2**	**±2**	**±4**	**±1**	**±2**	**±2**	**±4**	**±31**	**±31**
**Informed consent**	✓											
**Fulfilment of inclusion/exclusion criteria**	✓	✓				✓						
**Medical /medication history**	✓											
**Negative urine pregnancy test***		✓				✓						
**Haematology (screening)**	✓		✓			✓	✓			✓		
**Biochemistry (Screening)**	✓		✓			✓	✓			✓		
**Immunology (Screening)**	✓					✓				✓		
**Antibody studies (Trial sample)**		✓	✓	✓	✓	✓	✓	✓	✓	✓	✓	✓
**T- and B- responses (Trial sample)**		✓	✓	✓	✓	✓	✓	✓	✓	✓	✓	✓
**Randomisation**	✓											
**Vaccination**		✓				✓						
**Observation period (45 minutes)**		✓				✓						
**Examine vaccination site (45 minutes)**		✓				✓						
**Collection of Adverse Events**		✓	✓	✓	✓	✓	✓	✓	✓	✓		
**Serious Adverse Events**	**To be collected at any time during the trial**											

* for women only

The following blood samples will be collected:

Biochemistry screening (3.5mL SST™blood sample)

Haematology screening (3.mL EDTA blood sample)

Immunology screening (3.5mL SST™ blood sample)

Antibody studies (2 x 8mL clotted blood sample)

T- and B- cellular responses (3 x 8mL CPT™ heparinised)

Figure 5.2. Flow chart of the trial.

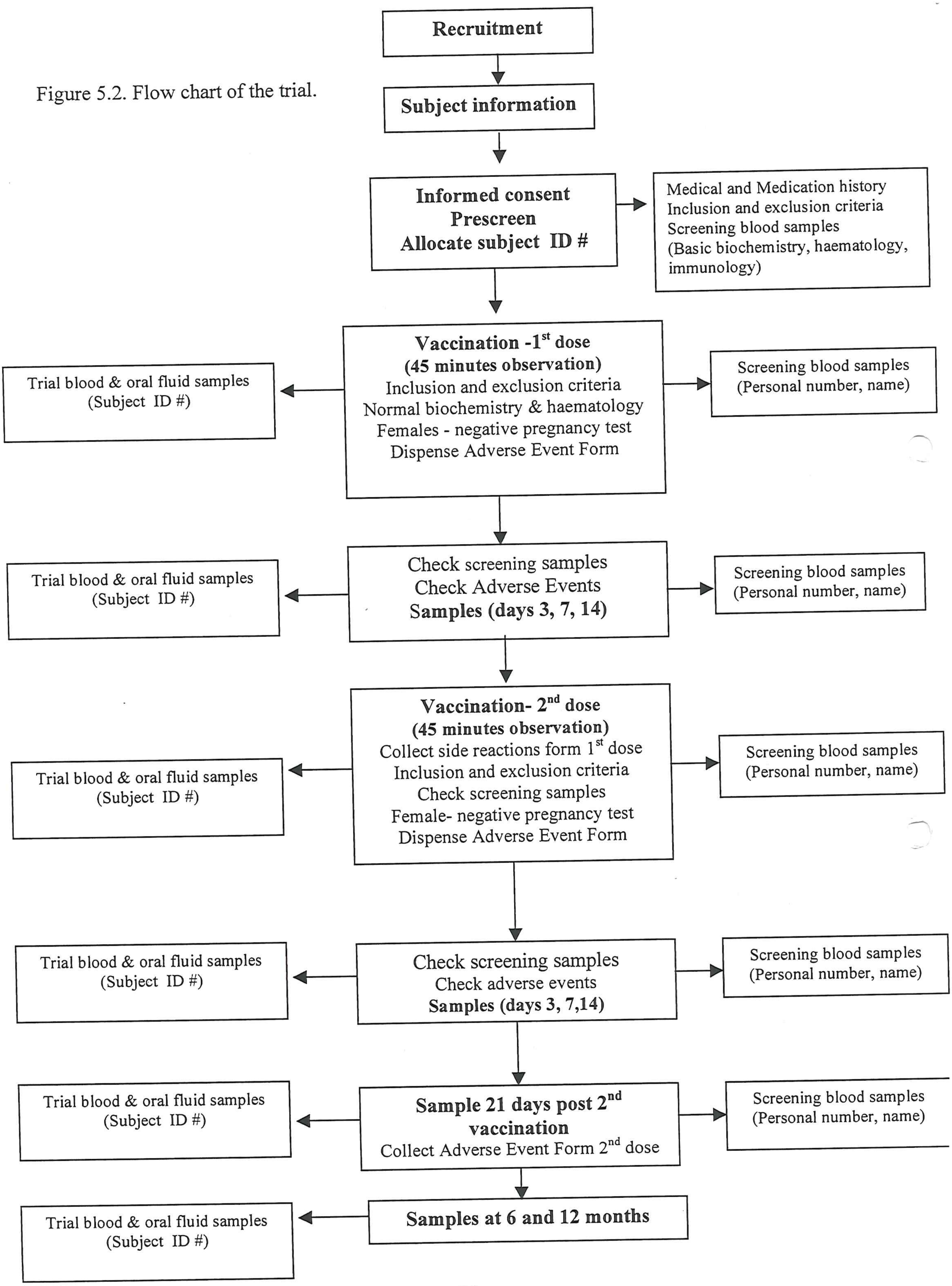

STUDY PROCEDURE

First visit: Day -14 (±12 days): Pre-screening

Subjects will be enrolled following one to one interviews providing they meet inclusion and not meeting the exclusion criteria and give fully signed informed consent. The following procedures will then be carried out and recorded in the Case Report Form:

- Record personal details including age, sex, personal number, next of kin;
- Record relevant medical and medication history;
- Record influenza vaccination history;
- Collect blood for haematological, biochemical, and immunological screening;
- Allocate a unique subject identification number;
- Schedule the next visit.

The following haematological, biochemical and immunological screening tests will be carried out:

- **Haematology:** red blood cell count, white blood cell count, % of neutrophils, eosinophils, basophils, monocytes, lymphocytes, platelets, haemoglobin haematocrit, mean cell volume
- **Biochemical parameters:** CRP, transaminases (ASAT, ALAT), alkaline phosphatase, Gamma GT, total bilirubin, total protein, albumin, glucose, blood creatinine, blood urea
- **Immunology tests:** ANA antibodies

Second visit: Day 0: First vaccination

Female volunteers will be tested for pregnancy using a urinary pregnancy test, and only included in the study upon a negative pregnancy test.

- The results of screening tests will be reviewed to determine if a subject has to be withdrawn
- Trial blood samples will be collected by venipuncture to measure the antibody, T- and B-cellular and antibody responses.
- An Orasure fluid sample will be collected to measure the local antibody response. The oral fluid sample will be collected using an absorbent pad placed on the gum to absorb the oral fluid for two minutes.
- Subjects meeting the inclusion and not meeting the exclusion criteria will be vaccinated with the appropriate vaccine parenterally (into the deltoid muscle of the upper arm (the

opposite side to that used for venipuncture)). The vaccine group and the side of injection will be recorded in the CRF

- Subjects will be observed for 45 minutes to check for immediate adverse reactions. Emergency equipment (adrenaline, corticosteroids) will be available in the event of a rare anaphylactic reaction to vaccine administration.
- The vaccination site will be examined for local reactions at the end of 45 minutes. These findings and any systemic adverse reactions will be recorded on the appropriate CRF page.
- Dispense the subject's adverse event forms to the subjects, and give instructions for its completion. The adverse event form must be returned to the site at the Day 21 visit.
- Each subject will be instructed in the evaluation of local and systemic adverse events.
- Schedule the next clinic visit.

Third visit: Day 3 (±1 day) post 1st vaccination

- The local and systemic reactions will be assessed; occurrence of adverse events, and use of analgesic or antipyretic medications will be recorded.
- Screening and trial blood samples will be collected by venipuncture. Trial blood samples will be collected to measure the antibody, T- and B- cellular and local antibody responses, respectively. The adverse event form will be reviewed.
- Schedule the next clinic visit.

The following haematological and biochemical screening tests will be carried out:

- **Haematology:** red blood cell count, white blood cell count, % of neutrophils, eosinophils, basophils, monocytes, lymphocytes, platelets,
- **Biochemical parameters:** CRP

Fourth visit: Day 7 (±2 day) post 1st vaccination

- The results of the screening samples will be reviewed.
- The adverse event form will be reviewed.
- Trial blood samples will be collected by venipuncture to measure the antibody, T- and B-cellular and antibody responses.
- An Orasure fluid sample will be collected to measure the local antibody response.
- Schedule the next clinic visit.

Fifth visit: Day 14 (±2 days) post 1st vaccination

- The adverse event form will be reviewed.
- Trial blood and Orasure samples will be collected to measure the antibody, T- and B-cellular and local antibody responses respectively.
- Schedule the next clinic visit.

Sixth visit: Day 21 (±4 days): Second Vaccination

- Collect adverse event form.
- Obtain interim history, including post injection reactions, adverse events and use of prescription and relief medication
- The results of the screening samples will be reviewed.
- Screening and trial blood will be collected by venipuncture.
- Collect Orasure samples for measuring the local antibody response.
- Female volunteers will be tested for pregnancy using a urinary pregnancy test, and only given the second dose of vaccine upon a negative pregnancy test.
- Subjects meeting the inclusion and not meeting the exclusion criteria will be vaccinated parenterally with the appropriate vaccine (the same vaccine group as for the first injection). Parenteral vaccination will be into the deltoid muscle of the upper arm (opposite side to that used for venipuncture). The vaccine group and the side of injection will be recorded in the CRF
- Subjects will be observed for 45 minutes to check for immediate adverse reactions. Emergency equipment (adrenaline, corticosteroids) will be available in the event of a rare anaphylactic reaction to vaccine administration.
- Examine the site of injection of the vaccine for local reactions at the end of 45 minutes. These findings and any systemic reactions will be recorded on the appropriate CRF page.
- Dispense a new adverse event form to the subjects, and give instructions for its completion.
- Schedule the next clinic visit.

The following haematological, biochemical and immunological screening tests will be carried out:

- **Haematology:** red blood cell count, white blood cell count, % of neutrophils, eosinophils, basophils, monocytes, lymphocytes, platelets, haemoglobin haematocrit, mean cell volume

- **Biochemical parameters:** CRP, transaminases (ASAT, ALAT), alkaline phosphatase, Gamma GT, total bilirubin
- **Immunology tests:** ANA antibodies

Seventh visit: Day 3 (±1 day) post 2nd vaccination

- The local and systemic reactions, occurrence of adverse events, and use of analgesic or antipyretic medications will be assessed. Data collection will be recorded in the CRF.
- The results of the screening samples will be reviewed.
- Screening and trial blood samples will be collected by venipuncture. Trial blood and Orasure samples will be collected to measure the antibody, T- and B- cellular and local antibody responses, respectively.
- The adverse event form will be reviewed.
- Schedule the next clinic visit.

The following haematological and biochemical screening tests will be carried out:

- **Haematology:** red blood cell count, white blood cell count, % of neutrophils, eosinophils, basophils, monocytes, lymphocytes, platelets
- **Biochemical parameters:** CRP

Eight visit: Day 7 (±2 day) post 2nd vaccination

- The results of the screening samples will be reviewed.
- The adverse event form will be reviewed.
- Trial blood and Orasure samples will be collected to measure the antibody, T- and B- cellular and local antibody responses, respectively.
- Schedule the next clinic visit.

Ninth visit: Day 14 (±2 days) post 2nd vaccination

- The adverse event form will be reviewed.
- Trial blood and Orasure samples will be collected to measure the antibody, T- and B- cellular and local antibody responses, respectively.
- Schedule the next clinic visit.

Tenth visit: Day 21 (±4 days) post 2nd vaccination

During this visit the following will be carried out and recorded in the CRF;

- Collect and discuss Adverse Event Form.
- Obtain interim history, including post injection reactions, adverse events and use of prescription and relief medication
- Screening and trial blood samples will be collected by venipuncture.

The following haematological, biochemical and immunological screening tests will be carried out:

- **Haematology:** red blood cell count, white blood cell count, % of neutrophils, eosinophils, basophils, monocytes, lymphocytes, platelets, haemoglobin haematocrit, mean cell volume
- **Biochemical parameters:** CRP, transaminases (ASAT, ALAT), alkaline phosphatase, Gamma GT, total bilirubin, total protein, albumin, glucose, blood creatinine, blood urea
- **Immunology tests:** ANA antibodies

Eleventh visit: 6 months (±31 days) post 2nd vaccination

- Subjects will be asked to give a trial blood sample.
- An Orasure sample will be collected.

Twelfth visit: 12 months (± 31 days) post 2nd vaccination

- Subjects will be asked to give a trial blood samples.
- Orasure samples will be collected.

Laboratory Analysis

The trial blood samples will be collected at 0, 3, 7, 14 and 21 days after each dose of vaccine (as detailed below) and at 6-12 months post vaccination to be used in serological assays and to study the kinetics of the T- and B-cellular responses. The serological assays (HI, SRH and neutralisation assays) will be carried out at the reference laboratories the National Institute of Biological Standards and Control and Health Protection Agency, UK and Istituto Superiore di Sanità, Italy according to established standard operating procedures (SOP). The modified HI assay using horse erythrocytes will be used as it shows increase sensitivity over the conventional HI test for detection of H5N1 antibody [56]. The kinetics of the immune response elicited after vaccination

with a H5N1 vaccine will be conducted using blood and oral fluid samples at the University of Bergen. The detailed immunological response will be studied using a variety of immunological methods e.g. serum antibody (HI, ELISA for antibody class and IgG subclasses, neuraminidase assays), peripheral blood antibody secreting cells (e.g. ELISPOT and non secreted IgG from B cells), lymphocyte profiles (e.g. FACS analysis) and cytokine producing T-cells (e.g. ELISPOT, ELISA, multiplex analysis). The long lasting and cross reactive immunity will be studied (National Institute of Biological Standards and Control and Health Protection Agency, UK, Istituto Superiore di Sanità, University of Bergen) using established appropriate immunological assays e.g. ELISA, HI, SRH, or FACS analysis of lymphocytes stimulated *in vitro* with different H5 strains. Sera may also be used in other serological tests deemed appropriate for detection of influenza specific responses e.g. western blots, pseudotype assays and epitope mapping using the phage display system.

5. A description of the stopping rules for individuals, parts of trial and entire trial

The clinical investigator will evaluate the local and systemic adverse reactions of each subject. If a subject experiences serious unexpected local or systemic adverse reactions then he/she may be withdrawn from the study. The screening samples should be within the normal range. Subjects that have out of range values will be withdrawn from the trial if the responsible clinician believes it to be in their best interest. If a suspected unexpected serious adverse reaction (SUSAR) occurs further enrolment in the trial will be suspended until the SUSAR has been fully investigated and the sponsor and regulatory agency agree to continue enrolment.

6. Maintenance of trial treatment codes and procedures for breaking the codes.

This study is an open study but the serological samples will be coded so that only the clinical investigators are aware of the code. The code will be broken once the trial is completed and all the immunological assays, except 6 and 12-month samples, have been performed.

7. The identification of any data to be recorded directly in CRFs and to be considered to be source data

The study data will be verifiable to the source data, which necessitates access to all original recordings, such as subject list, blood sample log, vaccine administration log and adverse event

forms. A daily log will be kept of all events related to the trial. On the day of vaccination, each subject will be randomly allocated a unique subject identification number in order to protect confidentiality. The original source documentation will be a paper-based database, which will include the original hard copy of the results from the two computer databases (referred to below) of the screening samples and serological results, and other information related to clinical trial. All databases will be secured against unauthorised access and confidentiality will be maintained at all times. One paper database will be held at the immunisation clinic at the hospital and will contain personal data (name, sex, age, contact details, next of kin, and the National Personal Identification Number (personal number) and the screening test results. The screening test results will be stored in NetLab database and only the relevant Haukeland University Hospital laboratory staff will have access to this database, by use of his/her personal access code to the hospital database. The second database will be laboratory-based and contain the subject identification number and data relating to immunological responses, the only personal data will be age, sex and previous influenza history. The sponsor or her nominee will only have access to this database and it will be secured against unauthorised access.

6 Selection and Withdrawal of subjects

1. Subjects inclusion criteria

The following subjects will be considered eligible to enter the study or receive the second dose of vaccine:

- Healthy volunteers (as concluded from the medical history, physical examination, and clinical judgment) aged 19 to 50 years old
- Females using a reliable method of contraception (from 4 weeks prior to the first vaccination until 4 weeks after the second vaccination) and a negative urine pregnancy test will be required before administration of each dose of vaccine
- Signed informed consent
- Subjects able to understand and comply with the study protocol and complete the Adverse Event Form
- Subjects able to attend the scheduled visits

- Subjects with normal pre-screening values. If a subjects prescreening samples lie outside the reference values he/she will only be included in the study based upon the medical evaluation of the clinical investigator

2. Subjects exclusion criteria

The following subjects will not be considered eligible to enter the study or receive the second dose of vaccine:

- Persons with a history of anaphylaxis or serious reactions to any vaccine
- Persons with known hypersensitivity to any of the vaccine components
- Persons who have had a temperature >38°C during the previous 72 hours
- Persons who have had an acute respiratory infection during the last 7 days
- Women who are pregnant or breast-feeding
- Persons with chronic illness at any stage that could interfere with trial conduct or compliance
- Persons who have received blood products or immunoglobulins parenterally during the previous 3 months
- Persons who have been vaccinated with any vaccine during the 4 weeks preceding the first trial vaccination
- Persons with known or suspected immunosuppressive disease or who use systemic immunosuppressive drugs
- Persons taking immunostimulant therapy
- Persons involved in another clinical trial during the last month.
- Suspected non-compliance

3. Subjects withdrawal criteria

When and how to withdraw subjects from the trial

The clinical investigator will evaluate the local and systemic adverse reactions of each subject. If a subject experiences unexpected serious local or systemic reactions then he/she may be withdrawn from the study. The screening samples should be within the normal range (see Table 6.1). Subjects that have out of range values will be withdrawn from the study, if the responsible clinician believes it to be in their best interest.

The type and timing of data to be collected for withdrawn subjects

The subjects who are withdrawn by the clinical investigator from the trial will continue to receive the clinically necessary screening tests as scheduled in the trial protocol. The clinical investigator will also conduct more frequent tests if deemed clinically necessary. No further trial samples will be collected from subjects who withdraw their informed consent for their participation in the study.

Whether and how subjects are to be replaced

Subjects will not be replaced if they are withdrawn or voluntarily withdraw from the trial

The follow-up for subjects withdrawn from treatment

This study is a vaccine study and subjects who are withdrawn will not receive the second dose of vaccine, if they are withdrawn after the first dose. The subjects who are withdrawn from the trial will continue to receive the screening tests, if clinically necessary, as described in the protocol and if deemed clinical necessary these tests will be carried out more frequently.

7. Treatment of Subjects

1. Treatment to be administered including name of all products, the doses, the dosing schedule, the route of administration and the treatment periods including follow-up periods for the subjects for each product treatment group.

Sixty healthy subjects aged 19-50 years who will receive two doses of H5N1 influenza vaccine by intramuscular injection into the deltoid muscle, separated by 21 ± 4 days. Escalating doses will be separated by a period of one week. Four groups of 15 subjects will receive two doses of pandemic virosomal A/H5N1 influenza vaccine containing

Group 1	30μg HA IM,
Group 2	1.5μg HA adjuvanted with 50μg 3rd generation ISCOM™ IM,
Group 3	7.5μg HA adjuvanted with 50μg 3rd generation ISCOM™ IM,
Group 4	30μg HA adjuvanted with 50μg 3rd generation ISCOM™ IM.

The second vaccine dose will contain the same quantity of antigen and adjuvant as in the first dose. Each subject will be enrolled in the trial for 1 year. They will be involved in the trial for a period of 10 weeks, during which blood samples will be collected at days 0, 3, 7, 14 and 21 days after each dose of vaccine. All subjects will be asked to voluntarily provide trial blood samples at 6 and 12 months after first dose of vaccine to examine the longevity of the antibody response.

Subjects will be pre-screened to ensure that they meet the entry criteria, and screened during the study for haematological, biochemical and immunological parameters to evaluate any systemic adverse events reactions.

The following parameters will be studied

- **Haematology:** red blood cell count, white blood cell count, % of neutrophils, eosinophils, basophils, monocytes, lymphocytes, platelets, haemoglobin haematocrit, mean cell volume
- **Biochemical parameters:** CRP, transaminases (ASAT, ALAT), alkaline phosphatase, Gamma GT, total bilirubin, total protein, albumin, glucose, blood creatinine, blood urea,
- **Immunology tests:** ANA antibodies

2. The methods and timing of the screening tests

Subjects will be pre-screened for haematological, biochemical and immunological tests to ensure they meet the inclusion criteria for the study. During the study, haematological, biochemical and immunological tests will be conducted to ensure that the vaccine is well tolerated. Blood samples will be collected at day 3 to examine for acute reactions and at 21 days after 1st and 2nd dose of vaccine to examine for longer term adverse events as detailed in the Table 9.1. The normal values for the screening samples are shown in Table 9.2.

Table 9.1 The timing of the screening tests

Timing of samples	Haematology	Biochemistry	Immunology
Pre-screen, days 0, 3, 21 after each dose of vaccine	red blood cell count<br>white blood cell count<br>(% of neutrophils, eosinophils, basophils, monocytes, lymphocytes)<br>platelet count	CRP	
Pre-screen, 21 days after 1st and 2nd vaccine dose	haemoglobin<br>haematocrit<br>mean cell volume	transaminases (ASAT, ALAT)<br>alkaline phosphatase<br>gamma GT<br>total bilirubin	ANA antibodies
Pre-screen, 21 days after 2nd vaccine dose		total protein<br>albumin<br>glucose<br>blood creatinine<br>blood urea<br>LD	

Table 9.2 Normal reference ranges for the screening tests.

**Haematological parameters**	**Reference ranges**
red blood cell count	3.7-5.8 10^{12}/L
white blood cell count	3.5-11.0 10^9/L
neutrophils	1.7-8.2 10^9/L
eosinophils	0.0-0.7 10^9/L
basophils	0.0-0.3 10^9/L
monocytes	0.04-1.30 10^9/L
lymphocytes	0.7-5.3 10^9/L
platelets	145-348 10^9/L
haemoglobin (female)	11.6-16.0 g/dL
haemoglobin (male)	13.2-16.6 g/dL
haematocrit	0.40-0.50 1
mean cell volume (MCV)	80-102 fL
**Biochemical parameters**	
CRP	<10 mg/L
ASAT	15-45 U/L
ALAT	10-70 U/L
alkaline phosphatase	35-105 U/L
Gamma GT	15-115 U/L
total bilirubin	5-25 μmol/L
total protein	62-78 g/L
albumin	36-45 g/L
glucose	4.0-6.0 mmol/L
blood creatinine	60-105μmol/L
blood urea	2.6-8.1 mmol/L
**Immunology tests**	
ANA antibodies	<0.99 (ratio)

3. Medication permitted (including rescue medication) and not permitted before and /or during trial

All subjects enrolled in this trial may continue their normal medication throughout the study. Subjects may continue to take therapy for chronic medical conditions, but must be maintained on a stable regimen for at least 2 weeks prior to study entry as assessed by their Medical History. However, subjects taking medication for malignant diseases or medication which may cause immunosuppression, will be excluded from enrolling in this study.
If anaphylactic shock occurs after vaccination, it will be treated according to the standard routines at Haukeland University Hospital using hydrocortisone sodium succinate (Solu-Cortef 100mg), dexchlorfeniramine (5mg/mL), atropine (1mg/ml), adrenaline (1mg/mL and 0.1mg/mL). Use of other medication including over-the-counter products will be discouraged during the study. Investigational drugs are prohibited during the course of the study.

4. Procedures for monitoring subject compliance

This study is a vaccine study and the daily log of events and CRF will be used to record if subjects have been vaccinated and provided the appropriate trial samples.

8 Assessment of Efficacy

1. Specification of efficacy parameters

In the absence of true efficacy markers like prevention of illness and/or reduction of morbidity and mortality, serum anti-HA antibodies are the most commonly measured correlate of protection against influenza [57] or surrogates of efficacy. The CHMP have defined criteria for the annual update for seasonal influenza vaccines, which measure the serological responses by the haemagglutination inhibition or single radial haemolysis assays. An HI titre ≥ 40 (or an equivalent SRH > $25mm^2$) indicates 50% protective levels of serum antibody against influenza infection in man. The EU evaluation criteria in groups of at least 50 adults aged 18-60 approximately at 3 weeks after vaccination are:

- The number of seroconversions or significant increases in anti-haemagglutinin antibody titre should be > 40%;
- Mean geometric increase >2.5;

- The proportion of subjects achieving an HI titre ≥40 or SRH > 25mm^2 should be >70%

The CHMP require for marketing authorisation of a pandemic vaccine that the vaccine fulfils all current evaluation criteria despite the immunological naivety of the host. The serological responses of the four vaccine groups will be evaluated and the ability of each group to meet the CHMP criteria will be determined. The ability of the different vaccine strengths to elicit neutralising antibodies will also be investigated.

In a pandemic situation, it is important to know how rapidly the immune response is elicited after vaccination with a novel subtype [52]. Thus, the detailed kinetics of the immune response in immunologically naïve individuals will also be assessed through the induction of local and systemic antibody and cellular immune responses and results will be compared to published results on the response to current influenza vaccines in healthy adults. Furthermore, there is a clear need for improved understanding of the immune response elicited in human volunteers including the quality and repertoire of the antibody response which will be evaluated by ELISA and phage display, respectively. Two other important factors are the capacity of the vaccine to elicit long lasting immunity and cross reactive immunity to H5 influenza viruses [52]. These will be evaluated by the standard serological assays and through the induction of local antibody and systemic cellular immune responses.

2. Methods and timing for assessing recording and analyzing efficacy parameters

Blood samples will be taken for serological assays at days 0, 3, 7, 14 and 21 after 1st and 2nd doses of vaccine. This study is an open study but all sera will be coded and blinded assays will be conducted by National Institute of Biological Standards and Control, Health Protection Agency and Istituto Superiore di Sanità, (SRH, HI, and neutralisation assays) on samples collected up to 21 ± 4 days after second dose of vaccine. These assays will be run with the international human anti- H5N1 standard plasma pool to allow comparison with other H5N1 pandemic clinical trials. When these serological assays are completed the code will be broken and the results sorted into appropriate vaccine group. The influence of the use of adjuvant and different antigen strengths on the immunogenicity of a virosomal A/H5N1 influenza vaccine on each of the sampling days and particularly 21 days post vaccination will then be assessed according to the three evaluation criteria defined by the CHMP. For each vaccine group the following will be calculated:

- The number of subjects who seroconvert or show significant increases in anti-haemagglutinin antibody titre;
- The mean geometric increase;
- The proportion of subjects achieving an HI titre ≥ 40 or SRH > $25mm^2$.

The immunogenicity of the vaccine will also evaluated by

- Producing a detailed time course of the immune response to vaccination including the kinetics of the T, B- and serum antibody responses
- The ability of the vaccine to induce long lasting immunity using the blood samples collected at 6 and 12 months after vaccination.
- The ability of the vaccine to induce cross-reactive immunity to other H5 strains.
- Examining the quality and repertoire of the antibody response.

Deviation from protocol

The investigators will adhere and try to avoid deviations from the protocol. The clinical investigators will document and explain any deviations from the approved protocol. Blood sampling for kinetic studies (T, B and antibody) can be collected within the following time frames after both the first and second doses of vaccine – 14 ± 12 days (pre-screening) and after the first vaccination; day 0 ± 0, 3 ± 1, 7 ± 2, 14 ± 2, 21 ± 4. Subjects who do not adhere to these sampling time points have deviated from the protocol and their inclusion in the comparative serological analyses will be evaluated by the investigators.

Protocol amendments

The sponsor will treat any protocol amendments according to European guideline "Detailed guidance for the request for authorisation of a clinical trial on a medicinal product for human use to the competent authorities, notification of substantial amendments and declaration of the end of the trial". The regulatory authorities will be notified about substantial amendments and these amendments must be approved before being implemented.

9 Assessment of Safety

1. Averse events (AE)

Note that the term IMP refers to the pandemic virosomal A/H5N1 influenza vaccine in this study.

Definitions

Adverse events

An AE is any untoward medical occurrence in a patient or subject which does not necessarily have a causal relationship with a medical treatment. This includes any noxious, pathological or unintended change in anatomical, physiological, or metabolic functions as indicated by physical signs, symptoms and/or laboratory-detected changes. These might occur in any phase of the clinical study whether associated with or related to the IMP or not. This includes also exacerbations of pre-existing conditions or events, intercurrent illnesses, or vaccine- or drug interactions. Anticipated day-to-day fluctuations of pre-existing conditions that do not represent a clinically significant exacerbation are not considered AEs. Discrete episodes of chronic conditions occurring during a study period should be reported as AE in order to assess changes in frequency or severity.

Serious adverse events (SAE)

SAEs are a subset of AEs. A SAE is defined as any untoward medical occurrence or effect at any dose that

- results in **death**
- is **life-threatening**: i.e. the subject was at risk of death at the time of the event; it does not refer to an event which hypothetically might have caused death if it were more severe
- results in persistent or significant **disability/incapacity**: i.e. results in a substantial disruption of the subject's ability to carry out normal life functions.
- requires in-patient **hospitalisation** or prolongation of existing hospitalisation: i.e. the subject is detained (usually at least an overnight stay) at the hospital or emergency ward for treatment that would not have been appropriate in the physician's office or in an out-patient setting. Hospitalisation for either elective surgery related to a pre-existing condition which did not increase in severity or frequency following initiation of the study or for routine clinical procedures[1] (including hospitalisation for "social" reasons) that are not the result of an AE are **not** considered as SAEs

---

[1] A procedure which may take place during the study period and should not interfere with the IMP administration or any of the ongoing protocol-specific procedures.

- is a **congenital anomaly or birth defect** in the offspring of a study subject
- is an **important medical event** that may jeopardise the subject or may require intervention to prevent one of the other outcomes listed above: e.g. interventions such as intensive treatment in an emergency room or at home for allergic bronchospasm; blood dyscrasias or convulsions that do not result in hospitalization, or development of drug dependency or drug abuse. Based on medical and scientific judgment, these events should usually be considered serious

Although **not** considered as SAE, cancer should be reported in the same way as SAEs.
Note: If anything untoward is reported during an elective procedure, that occurrence must be reported as an AE, either serious or non-serious according to the criteria defined above. When in doubt as to whether hospitalisation occurred or was necessary, the AE should be considered serious.

Surveillance, reporting, and documentation of adverse events

The recording of AEs is an essential part of study documentation. The clinical investigator is responsible for documenting all AEs as set out in the following sections.
Documentation of solicited adverse events

Solicited AEs are precisely defined events that the subjects are specifically asked about and which are documented by the subjects in the adverse event form. This form will be the source document and will be added into the CRF.

The following local AEs at the IMP injection site (injections site reactions) will be documented in the Adverse Event Form:

- Pain
- Erythema
- Ecchymosis
- Induration
- Itching
- Swelling

The following solicited systemic AEs will be documented in the adverse event form and transcribed by the investigator into the CRF:

- Body temperature (oral) ≥ 38°C
- Malaise
- Shivering
- Fatigue
- Headache

- Sweating
- Myalgia
- Arthralgia
- Diarrhoea
- Respiratory symptoms

An Adverse Event Form will be issued to all subjects instructing them to record their daily local and systemic adverse events for as long as symptoms persist. The subjects will provide information on the presence or absence of solicited local and systemic adverse events. The use of antipyretic/relief medication and any changes in medication will also be recorded. Subjects will score the severity of symptoms as follows:

Blank – no adverse symptoms

1 -symptom occurred but not severe enough to cause inconvenience

2 -symptom occurred severe enough to interfere with daily activities but required minimal or no medical intervention

3-symptom occurred severe enough to markedly interfere with daily activities; required medical consultation

At each visit, all AEs, either observed by the clinical investigator or his nominee or reported by the subjects spontaneously or in response to a direct question will be evaluated by the investigator. As a consistent method to find out about adverse events, the investigator should use a non-leading question, such as:

"Have you felt different since receiving the vaccine or since the previous visit?"

The subjects will be instructed to contact the clinical investigator immediately should they experience any signs or symptoms they perceive as serious during the period extending from the first study-specific procedure up to and including 6 months (minimum 180 days) after the last administration of the IMP.

Documentation of adverse events

Any AE occurring within 3 weeks (minimum 17 days) following vaccination will be recorded on the AE Page of the CRF, irrespective of its severity or potential relationship to the IMP.

Any SAE brought to the attention of the investigator from study start up to 6 months (at least 180 days) after vaccination of that subject will be recorded. AEs not previously documented in the adverse event form will be recorded in the AE page of the CRF. AEs should be documented in terms of signs and symptoms observed by the investigator or reported by subjects. Whenever

possible, a medical diagnosis should be made. The nature of each event, date and (where appropriate) time of onset, outcome, severity and causal relationship should be established. Details of any symptomatic/corrective treatment should be recorded on the appropriate page of the CRF.

Hospitalisation for routine clinical procedures (including hospitalisation for "social" reasons) that are not the result of an AE are not considered AEs but will be recorded on the AE page of the CRF. The same applies for hospitalisation for elective procedures related to a pre-existing condition that did not increase in severity or frequency during the study. If hospitalisation was planned before first administration of the IMP, it will be documented in the Medical History Page of the CRF (see below).

The following events will be documented in the Medical History Page of the CRF:

- AEs which occur after informed consent was obtained, but before the first IMP administration
- Pre-existing conditions or signs and/or symptoms present in a subject before study start. This includes conditions which were not recognized at study entry but later during the study period
- Hospitalisation arising from a pre-existing condition and planned before the first administration of the IMP

Reporting of serious adverse events

All SAEs must be reported immediately by the investigator without filtration, independent of their association or causal relation to the IMP. The investigator must report all SAEs within 1 calendar day of becoming aware of the event by telephone, fax or e-mail (if appropriate) to the Study Contact for SAE Reporting as described below.

The initial notification should include all known information regarding the SAE (which may be minimal, but should include sufficient information to permit identification of the reporter, the subject, the IMP, the SAE, and the date of onset of the SAE). The investigator should not wait for additional information to fully document the event before notifying the Study Contact for SAE Reporting. A SAE Report Form should be filled out and sent to the Study Contact for SAE Reporting within 1 day of becoming aware of the event. The initial notification sent by the investigator will be confirmed by an acknowledgement letter. The initial report should then be followed by submission of a completed SAE Report Form provided by the sponsor as soon as possible but at latest within 3 calendar days of the initial report.

The completed SAE Report Form should detail all relevant aspects of the SAE, all actions taken by the investigator, the causality, and the outcome of the event. Any new information obtained regarding the SAE must be reported immediately. The SAE Report Forms should be used for documentation of the SAE, any actions taken, the outcome, and for follow-up reports. When applicable, hospital case records and autopsy reports should be obtained by the investigator and forwarded to the Study Contact for SAE Reporting.

The investigator must report SAEs to the appropriate Ethics Committee, if requested by the committee, and/or according to local legal requirements.

Study Contact for SAE Reporting:

Pharmacovigilance Department, Crucell Berna Biotech Ltd.

Tel: +41 31 980 64 29

Fax: +41 31 980 65 89

Outside office hours:

Fax: +41 31 980 65 89

E-mail: pharmacovigilance@crucell.com

The reporting obligations for SUSARs will be fulfilled as required by the applicable law.

Causality of adverse events

Every effort should be made by the investigator to explain any AE and assess its potential causal relationship to administration of the IMP. This applies to all AEs, i.e. to both non-serious and serious adverse events.

To which degree of certainty an AE can be attributed to administration of the IMP or to alternative causes (e.g. natural history of the underlying diseases, concomitant therapy) will be determined by how well the event can be understood in terms of one or more of the following:

- A reaction of a similar nature has been previously observed with this type of medicinal product and/or formulation
- The event has often been reported in literature for similar types of medicinal products
- The event has been temporally associated with IMP-administration or reproduced on re-administration of the IMP

By definition, all solicited local AEs occurring at the application site (i.e. all solicited local reactions) will be considered related to the IMP-administration (injection site reactions).

Causality of all other AEs should be assessed by the investigator based on the following:
In your opinion, is there a reasonable possibility that the AE was caused by the IMP?

Related	there is suspicion that there is a relationship between IMP and AE (without determining the extent of probability); there is a reasonable possibility that the IMP contributed to the AE
Unrelated	there is no suspicion that there is a relationship between IMP and AE; there are other more likely causes and administration of the IMP is not suspected to have contributed to the AE

Severity of adverse events

The severity of unsolicited AEs will be graded on a 3-point scale as follows:

**Grade**	**Definition**
1 (mild)	Discomfort noted, but no disruption of normal daily activity; slightly bothersome; relieved with or without symptomatic treatment.
2 (moderate)	Discomfort sufficient to reduce or affect normal daily activity to some degree; bothersome; interferes with normal daily activities; only partially relieved with symptomatic treatment.
3 (severe)	Discomfort sufficient to reduce or affect normal daily activity considerably; prevents regular activities; not relieved with symptomatic treatment.

Follow-up of ongoing adverse events and assessment of outcome

Follow-up of non-serious adverse events

Non-serious AEs already documented in the CRF at a previous assessment and designated as 'ongoing' should be reviewed at subsequent visits. If the event has resolved, the documentation in the CRF should be completed. If the frequency or severity of a non-serious AE changes significantly, a new record of the AE has to be started. If the AE becomes serious, the procedures for reporting of SAEs have to be followed (see above).

Ongoing non-serious AEs will be followed until the end of the active study phase.

Outcome will be assessed as:

1 Resolved (no sequelae)

2 Resolved (with sequelae)

3 Ongoing

4 Death

5 Unknown

Follow-up of serious adverse events

All SAEs must be followed-up until the event has either resolved, subsided, stabilized, disappeared, or is otherwise explained, or the study subject is lost to follow-up, but no longer than 6 months after the last vaccination.

All follow-up activities must be reported in a timely manner to the Study Contact for Reporting of SAEs (if necessary on one or several consecutive SAE report forms). All fields of the form with additional or changed information must be completed and the SAE Report Form should be forwarded to the Study Contact for SAE Reporting as soon as possible but at the latest within 7 calendar days after receipt of the new information. Clinically significant laboratory abnormalities reported as SAEs will be followed-up until they have returned to normal or a satisfactory explanation has been provided. Reports related to the subsequent course of any SAE reported for any subject must be submitted to the Pharmacovigilance Department Berna Biotech Ltd.

Treatment of adverse events

Treatment of any AE is at the sole discretion of the investigator and according to current available best treatment. The applied measures should be recorded in the CRF.

2. Handling of pregnancy cases

Subjects who become pregnant during the study period (up to and including 1 month [minimum 30 days] after receiving the last IMP dose) must not receive additional doses of IMP but may

continue other study procedures at the discretion of the investigator. Subjects should be instructed to notify the investigator if it is determined after completion of the study that they became pregnant either during the study or within 1 month (minimum 30 days) after receiving the vaccine dose.

A pregnancy should be followed to term, any premature terminations reported, and the health status of the mother and child including date of delivery, sex, and weight of the child, should be reported to the sponsor after delivery. Any abnormality considered related to the IMP by the responsible physician during pregnancy and delivery, and health of mother and baby must be reported through an expedited report.

10. Statistics

1. A description of the statistical methods to be employed

This is a phase I study in a limited number of subjects being conducted primarily to assess safety and immunogenicity, thus showing proof of concept that the vaccine is safe and immunogenic. Therefore, a larger phase II/III study would be required with higher statistical power to provide further safety and immunogenicity data.

2. The number of subjects planned to be enrolled and reason for choice of sample size

Sixty young healthy subjects (aged 19-50 years old) will be randomly assigned to one of four groups, each group will contain 15 subjects and the investigators will aim to have an equal distribution of males and females in each group and similar age distribution.

Deviation from protocol and procedures for accounting for missing data

The investigators will adhere and try to avoid deviations from the protocol. All screening and trial blood samples can be collected within the following time frames after both the first and second doses of vaccine – 14 ± 12 days (pre-screening) after the first vaccination; Day 0 ± 0, 3 ± 1; 7 ± 1, 14 ± 2, 21 ± 4. The clinical investigators will document and explain any deviations from the approved protocol. Subjects who do not adhere to these sampling time points have deviated from the protocol and their inclusion in the comparative analyses will be evaluated by the investigators.

11. Direct access to source data/documentation

The study data will be verifiable to the source data, which necessitates access to all original recordings, such as informed consent, subject list, serum sample log, vaccine administration log and Adverse Event Form. A daily log will be kept of all events related to the trial. The clinical investigators are allowed access to the subject's hospital medical records. The subjects will be informed of this and will be signing their agreement when giving informed consent

On the day of vaccination each subject will be randomly allocated a unique subject identification number in order to protect confidentiality. Two computer databases will be used. All original screening test results will be stored in the hospital's NetLab system and patient confidentiality will be maintained according to the hospital's established guidelines. It will only be accessed by the relevant hospital laboratory staff , by use of his/her personal access code to the hospital database. The second database will be laboratory-based and the only personal data will be age and sex, previous influenza history and data relating to immunological responses. This database will only be accessed by the Principal Investigator or her nominee and the entry point will only be identified by the subject identification number. However, two paper-based databases (one clinical database and one laboratory based database) will be established and they will be considered as the original source documentation. The paper-based databases will include, the original hard copy of the documents from computer databases (e.g. results of screening samples and serological results), CRFs and other information related to clinical trial. One database will be held at the immunisation clinic at the hospital and will contain personal data (name, sex, age, contact details, next of kin, and the National Personal Identification Number (Personal number) and screening results). All databases will be secured against unauthorised access and confidentiality will be maintained at all times. Monitors, Auditors, Inspectors, and Regulatory Authorities will have access to the paper database if required.

12 Quality Control and Quality Assurance

All the data will be generated, recorded and reported under restricted quality assured system in accordance with GCP requirements. The principle investigator will assign a qualified person to double check and quality control (according to established SOPs) all data entries to ensure that they are correct and complete. Any changes, corrections or amendments in the original data and

records will be signed and dated by the authorized person. Furthermore, the changes will be conducted in a proper way to guarantee the traceability of the original data. All the source data, reports and documents will be accessible for internal auditing and to inspection by regulatory authorities.

13. Ethics

The PANFLUVAC consortium will investigate a virosomal H5N1 influenza vaccine for parenteral administration. This phase I study will provide initial proof of concept that the vaccine is safe and immunogenic. Influenza H5N1 has infected 387 people and results in 245 deaths, thus the volunteers may be protected against the investigated influenza H5N1 strain if it acquired the ability for human to human spread and became pandemic. The subjects will be clearly informed that there are no direct health benefits in the subject information sheet and upon enrolment in the trial. The benefit to the community is substantial, as we will gain knowledge about the safety and immunogenicity of a potential pandemic vaccine, which will aid in developing effective vaccination strategies for pandemic vaccines. The annual mortality in the EU due to influenza epidemics is estimated at 400 deaths per million people, but an influenza pandemic could increase mortality by a factor of ten. The knowledge gained by our study could considerably improve our ability to respond quickly in a pandemic scenario by producing a safe and effective vaccine, which should save many lives.

Good Clinical Practice

The study will be conducted according to the Declaration of Helsinki, ICH / Guidelines on Good Clinical Practice for Trials on Medicinal Products in the European Community and local legal requirements.

Regulatory authority approval

Regulatory authority approval will be obtained from the Norwegian Medicines Agency.

Ethics committee approval

The principal investigator will submit the protocol, subject information, informed consent, and

other study-related documents as required by applicable laws and regulations for approval to the relevant independent ethics committee (Regional komité for medisinsk forskningsetikk, Nord-Norge (REK Nord)) before study commencement. The ethics committee will be informed of all subsequent protocol amendments and of SUSARs occurring during the trial. The trial will not commence until approved by all relevant authorities.

Biobank and Data law

The relevant permission will be obtained from the Biobanken and Personvernombudet for forskning ved NSD before the start of the trial.

Patient informed consent

Prior to entry, the investigators will inform the volunteers of the purpose, nature, possible benefits and potential hazards of the study including any discomfort or adverse event which may occur. Subjects will be given the necessary time to ask for further information or clarification. The subject will then be given suitable time to decide if he or she wishes to participate in the trial. The subject can at any time point withdraw his/her consent, without detriment, and all subjects will be offered seasonal influenza vaccine for the upcoming influenza season after completion of the trial. No financial incentives will be offered. Each subject will provide a signed informed consent prior to any study-related activities.

Removal/withdrawal of subjects from treatment or assessment

Subjects may at any time withdraw their informed consent for their participation in the study without any resulting detriment. If a subject withdraws from this trial he/she will not be replaced. The clinical investigator may also withdraw a subject if it is believed to be medically in the best interest of the subject or if the subject cannot comply with the protocol.

Notification of primary care physician

The clinical investigator will inform the subject's general practitioner of their patient's participation in this clinical trial.

14 Data handling and record keeping

The clinical investigators will be responsible that data reported in the CRF and the adverse event forms are complete, accurate and legible record for each subject. The clinical investigators will also ensure that all data reported on the CRF is consistent with the original hospital records and that any corrections are appropriately dated and initialled. The clinical investigators will provide adequate instructions on completion of the adverse event form to each subject.

Storage and retention of study documentation

The documentation for this study will be stored as hard copy documents in a secure office where only the sponsor or his nominee has access to as required by GCP guidelines. The documents will be retained for a minimum of 15 years.

15 Financing and Insurance

The sponsor of this trial is the University of Bergen on behalf of the EU PANFLUVAC Consortium. The EU has provided the basic financing allowing production of the vaccine and its preclinical testing. The trial will be funded by the EU, the Influenza Centre, University of Bergen and Helse Vest.

If a subject is harmed by his/her participation in this clinical trial, he/she will be compensated according to established guidelines. The investigators have purchased a drug liability insurance policy with the Drug Liability Association (Legemiddelansvarsforeningen) which will insure all subjects in this trial according to Produktansvarsloven 23rd December 1988 nr. 104 chapter 3.

16 Publication Policy

The results of this study will be published in international peer reviewed journals. A substantial amount of information will be generated by the study. Publications arising out of the work will focus on:

- The comparative reactogenicity of escalating doses of H5N1 vaccines in an immunologically naïve population.

- The kinetics of the antibody and B and T cellular responses
- Evaluation of the ability of non-adjuvanted and 3rd generation ISCOM™ adjuvanted virosomal H5N1 influenza vaccine to fulfil the CHMP criteria
- Detailed time course of the immune response to vaccination with a novel H5N1 influenza subtype including the kinetics and quality of the serum antibody and T and B cellular responses and epitope mapping
- The longevity of the immunity induced by the vaccine
- The ability of the vaccine to elicit cross reactive immunity against other H5 strains.

17 Supplements

18 References

1. CHMP. Guideline on dossier structure and content for pandemic influenza. Vaccine marketing authorisation application. EMEA/CPMP/VEG/4717/03-Rev. 1 2008

2. Drane D, Pearse M. The ISCOMATRIX™ adjuvant. In: Schijns V, O'Hagan D, eds. Immunopotentiators in Modern Vaccines.: Elsevier Academic Press, 2006:191-213

3. Drane D, Gittleson C, Boyle J and Maraskovsky E. ISCOMATRIX adjuvant for prophylactic and therapeutic vaccines. Expert Rev Vaccines 2007;6:761-72

4. Belshe RB. The origins of pandemic influenza--lessons from the 1918 virus. N Engl J Med 2005;353:2209-11

5. World Health Organization. Cumulative Number of Confirmed Human Cases of Avian Influenza A/(H5N1) Reported to WHO. Vol. 2008, 2008

6. Cox RJ, Brokstad KA and Ogra P. Influenza virus: immunity and vaccination strategies. Comparison of the immune response to inactivated and live, attenuated influenza vaccines. Scand J Immunol 2004;59:1-15

7. Wood JM, Robertson JS. Reference viruses for seasonal and pandemic influenza vaccine preparation. Influenza and Other Respiratory Viruses 2007;1:5-9

8. Treanor JJ, Campbell JD, Zangwill KM, Rowe T and Wolff M. Safety and immunogenicity of an inactivated subvirion influenza A (H5N1) vaccine. N Engl J Med 2006;354:1343-51

9. Treanor JJ, Wilkinson BE, Masseoud F, et al. Safety and immunogenicity of a recombinant hemagglutinin vaccine for H5 influenza in humans. Vaccine 2001;19:1732-7

10. Stephenson I, Nicholson KG, Wood JM, Zambon MC and Katz JM. Confronting the avian influenza threat: vaccine development for a potential pandemic. Lancet Infect Dis 2004;4:499-509

11. Haaheim LR. Vaccines for an influenza pandemic: scientific and political challenges. Influenza and Other Respiratory Viruses 2007;1:55-60

12. Keitel WA, Atmar RL. Preparing for a possible pandemic: influenza A/H5N1 vaccine development. Curr Opin Pharmacol 2007;7:484-90

13. Madhun A, Haaheim L, Nilsen M and Cox R. High frequencies of polyfunctional Th1 CD4 cells and a strong antibody response were induced in mice after vaccination with parenteral virosomal H5N1 vaccine adjuvanted with ISCOM. Third European Influenza Conference. Vilamoura, Portugual, 2008

14. Ennis FA, Cruz J, Jameson J, Klein M, Burt D and Thipphawong J. Augmentation of human influenza A virus-specific cytotoxic T lymphocyte memory by influenza vaccine and adjuvanted carriers (ISCOMS). Virology 1999;259:256-61

15. Rimmelzwaan GF, Baars M, van Amerongen G, van Beek R and Osterhaus AD. A single dose of an ISCOM influenza vaccine induces long-lasting protective immunity against homologous challenge infection but fails to protect Cynomolgus macaques against distant drift variants of influenza A (H3N2) viruses. Vaccine 2001;20:158-63

16. Davis ID, Chen W, Jackson H, et al. Recombinant NY-ESO-1 protein with ISCOMATRIX adjuvant induces broad integrated antibody and CD4(+) and CD8(+) T cell responses in humans. Proc Natl Acad Sci U S A 2004;101:10697-702

17. Frazer IH, Quinn M, Nicklin JL, et al. Phase 1 study of HPV16-specific immunotherapy with E6E7 fusion protein and ISCOMATRIX adjuvant in women with cervical intraepithelial neoplasia. Vaccine 2004;23:172-81

18. Palache AM, vd Velden JW. Influenza vaccination in asthma. Lancet 1992;339:741

19. Beyer WE, Palache AM, de Jong JC and Osterhaus AD. Cold-adapted live influenza vaccine versus inactivated vaccine: systemic vaccine reactions, local and systemic antibody response, and vaccine efficacy. A meta-analysis. Vaccine 2002;20:1340-53

20. Nichol KL. Efficacy/clinical effectiveness of inactivated influenza virus vaccines in adults. In: Nicholson KG, Webster RG and Hay AJ, eds. Textbook of influenza. first ed. Oxford: Blackwell science, 1998:358-372
21. vaksinasjon Vo. Folkehelse 2007
22. Lasky T, Terracciano GJ, Magder L, et al. The Guillain-Barre syndrome and the 1992-1993 and 1993-1994 influenza vaccines. N Engl J Med 1998;339:1797-802
23. Askonas BA, Lin YL. An influenza specific T-killer clone is restricted to H-2Ld and cross-reacts with Dk region. Immunogenetics 1982;16:83-7
24. Cox RJ, Brokstad KA, Zuckerman MA, Wood JM, Haaheim LR and Oxford JS. An early humoral immune response in peripheral blood following parenteral inactivated influenza vaccination. Vaccine 1994;12:993-9
25. Powers DC, Smith GE, Anderson EL, et al. Influenza A virus vaccines containing purified recombinant H3 hemagglutinin are well tolerated and induce protective immune responses in healthy adults. J Infect Dis 1995;171:1595-9
26. Brokstad KA, Cox RJ, Olofsson J, Jonsson R and Haaheim LR. Parenteral influenza vaccination induces a rapid systemic and local immune response. J Infect Dis 1995;171:198-203
27. Brokstad KA, Cox RJ, Oxford JS and Haaheim LR. IgA, IgA subclasses, and secretory component levels in oral fluid collected from subjects after parental influenza vaccination. J Infect Dis 1995;171:1072-4
28. El-Madhun AS, Cox RJ and Haaheim LR. The effect of age and natural priming on the IgG and IgA subclass responses after parenteral influenza vaccination. J Infect Dis 1999;180:1356-60
29. el-Madhun AS, Cox RJ, Soreide A, Olofsson J and Haaheim LR. Systemic and mucosal immune responses in young children and adults after parenteral influenza vaccination. J Infect Dis 1998;178:933-9
30. Drane DL, Osato SS. Using the neurobehavioral cognitive status examination as a screening measure for older adults. Arch Clin Neuropsychol 1997;12:139-43
31. Hobson D, Curry RL, Beare AS and Ward-Gardner A. The role of serum haemagglutination-inhibiting antibody in protection against challenge infection with influenza A2 and B viruses. J Hyg (Lond) 1972;70:767-77
32. Schild GC, Pereira MS and Chakraverty P. Single-radial-hemolysis: a new method for the assay of antibody to influenza haemagglutinin. Applications for diagnosis and seroepidemiologic surveillance of influenza. . Bull World Health Organ. 1975;52: 43-50
33. WHO. WHO strategic action plan for pandemic influenza 2007
34. Committee. Guideline On Dossier Structure And Content Of Marketing Authorisation Applications For Influenza Vaccines Derived From Strains With A Pandemic Potential For Use Outside Of The Core Dossier Context EMEA/CHMP/VWP/263499/2006. European Agency for the Evaluation of Medicinal Products, July 24, 2006 1996
35. Hehme N, Engelmann H, Kunzel W, Neumeier E and Sanger R. Pandemic preparedness: lessons learnt from H2N2 and H9N2 candidate vaccines. Med Microbiol Immunol (Berl) 2002;191:203-8
36. Nicholson KG, Colegate AE, Podda A, et al. Safety and antigenicity of non-adjuvanted and MF59-adjuvanted influenza A/Duck/Singapore/97 (H5N3) vaccine: a randomised trial of two potential vaccines against H5N1 influenza. Lancet 2001;357:1937-43
37. Stephenson I, Nicholson KG, Colegate A, et al. Boosting immunity to influenza H5N1 with MF59-adjuvanted H5N3 A/Duck/Singapore/97 vaccine in a primed human population. Vaccine 2003;21:1687-93
38. Stephenson I, Nicholson KG, Gluck R, et al. Safety and antigenicity of whole virus and subunit influenza A/Hong Kong/1073/99 (H9N2) vaccine in healthy adults: phase I randomised trial. Lancet 2003;362:1959-66

39. Lin J, Zhang J, Dong X, et al. Safety and immunogenicity of an inactivated adjuvanted whole-virion influenza A (H5N1) vaccine: a phase I randomised controlled trial. Lancet 2006;368:991-7
40. Leroux-Roels I, Borkowski A, Vanwolleghem T, et al. Antigen sparing and cross-reactive immunity with an adjuvanted rH5N1 prototype pandemic influenza vaccine: a randomised controlled trial. Lancet 2007;370:580-9
41. Ehrlich HJ, Muller M, Oh HM, et al. A clinical trial of a whole-virus H5N1 vaccine derived from cell culture. N Engl J Med 2008;358:2573-84
42. Bresson JL, Perronne C, Launay O, et al. Safety and immunogenicity of an inactivated split-virion influenza A/Vietnam/1194/2004 (H5N1) vaccine: phase I randomised trial. Lancet 2006;367:1657-64
43. Hehme N, Engelmann H, Kuenzel W, Neumeier E and Saenger R. Immunogenicity of a monovalent, aluminum-adjuvanted influenza whole virus vaccine for pandemic use. Virus Res 2004;103:163-71
44. Keitel WA, Campbell JD, Treanor JJ, et al. Safety and Immunogenicity of an Inactivated Influenza A/H5N1 Vaccine Given with or without Aluminum Hydroxide to Healthy Adults: Results of a Phase I-II Randomized Clinical Trial. J Infect Dis 2008;198:1309-1316
45. Bernstein DI, Edwards KM, Dekker CL, et al. Effects of adjuvants on the safety and immunogenicity of an avian influenza H5N1 vaccine in adults. J Infect Dis 2008;197:667-75
46. Stephenson I, Bugarini R, Nicholson KG, et al. Cross-reactivity to highly pathogenic avian influenza H5N1 viruses after vaccination with nonadjuvanted and MF59-adjuvanted influenza A/Duck/Singapore/97 (H5N3) vaccine: a potential priming strategy. J Infect Dis. 2005;191:1210-5
47. (IVR) WHOIfVR. Tables on the Clinical trials of pandemic influenza prototype vaccines. 2008
48. Ninomiya A, Imai M, Tashiro M and Odagiri T. Inactivated influenza H5N1 whole-virus vaccine with aluminum adjuvant induces homologous and heterologous protective immunities against lethal challenge with highly pathogenic H5N1 avian influenza viruses in a mouse model. Vaccine 2007;25:3554-60
49. Lipatov AS, Hoffmann E, Salomon R, Yen HL and Webster RG. Cross-protectiveness and immunogenicity of influenza A/Duck/Singapore/3/97(H5) vaccines against infection with A/Vietnam/1203/04(H5N1) virus in ferrets. J Infect Dis 2006;194:1040-3
50. Suguitan AL, Jr., McAuliffe J, Mills KL, et al. Live, attenuated influenza A H5N1 candidate vaccines provide broad cross-protection in mice and ferrets. PLoS Med 2006;3:e360
51. Mahmood K, Bright RA, Mytle N, et al. H5N1 VLP vaccine induced protection in ferrets against lethal challenge with highly pathogenic H5N1 influenza viruses. Vaccine 2008;26:5393-9
52. CHMP. Core SPC for pandemic influenza vaccines. EMEA/CHMP/VEG/193031/2004 2004
53. Zuckerman M, Cox R, Taylor J, Wood J, Haaheim L and Oxford J. Rapid immune response to influenza vaccination. Lancet 1993;342:1113
54. Brokstad KA, Cox RJ, Major D, Wood JM and Haaheim LR. Cross-reaction but no avidity change of the serum antibody response after influenza vaccination. Vaccine 1995;13:1522-8
55. el-Madhun AS, Cox RJ, Seime A, Sovik O and Haaheim LR. Systemic and local immune responses after parenteral influenza vaccination in juvenile diabetic patients and healthy controls: results from a pilot study. Vaccine 1998;16:156-60
56. Stephenson I, Wood JM, Nicholson KG and Zambon MC. Sialic acid receptor specificity on erythrocytes affects detection of antibody to avian influenza haemagglutinin. J Med Virol 2003;70:391-8
57. Cox NJ, Subbarao K. Influenza. Lancet 1999;354:1277-82

Appendix

Norwegian Case Report Form

*Deltager ID #*__________ *Kjønn*_________ *Alder*_________ *Initialer*___________

CASE REPORT FORM

Ansvarlig lege ______________________________

Avdeling ______________________________

Dato for innrullering i studien ______________________________

Relevant sykehistorie (kommentarer)______________________________

____

____

____

____

____

____

____

____

Faste medisiner______________________________

____

Tidligere vaksinert mot influensa (år)______________________________

Antatt influensa siste 12 måneder ______________________________

Bekreftet med laboratorieprøve (Ja/nei) ______________________________

DERSOM DELTAKEREN TREKKER SEG/ BLIR TRUKKET FRA STUDIEN:

dato ______________________________

årsak (bare hvis prosjektledelsen har bestemt at deltakeren må forlate studien) _____

____

____

____

____

____

*Deltager ID #*_________ *Kjønn*_________ *Alder*_________ *Initialer*__________

Prescreen

INKLUSJONSKRITERIER: Alle setninger må besvares med JA for at personen skal kunne delta i studien

	**Ja**	Nei
Signert informert samtykke		
Frisk		
Mellom 20 og 40 år		
Kvinner: Bruker en pålitelig prevensjonsmetode så lenge studien pågår (fra 4 uker før 1. vaksinasjon til 4 uker etter 2. vaksinasjon)		
I stand til å møte på oppsatte tidspunkt, og følge gitte instruksjoner		

EKSKLUSJONSKRITERIER: Alle setninger må besvares med NEI for at personen skal kunne delta i studien

	**Nei**	Ja
Tidligere anafylaktisk sjokk eller alvorlige komplikasjoner etter vaksinering		
Kjent allergi overfor innholdstoffer i vaksine		
Feber >38°C i løpet av siste 72 timer		
Akutt luftveissykdom siste 7 dager		
Kvinner: gravid eller ammende		
Sykdom som kan påvirke evnen til å gjennomføre deltagelse i studien		
Fått blodprodukter eller immunglobulin parenteralt siste 3 måneder		
Kjent eller mistenkt immunsviktsykdom		
Bruk av medisiner som svekker immunforsvaret		
Bruk av immunstimulerende medikamenter		
Vaksinert i en 4 ukers periode før første vaksinasjon		
Vært med på andre kliniske utprøvninger siste måneden		

*Deltager ID #*__________ *Kjønn*__________ *Alder*__________ *Initialer*__________

Åpne rubrikker skal merkes av med aktuell dato for hvert besøk. Grå rubrikker skal ikke fylles ut.

	forundersøkelse Dag -14 ±12	Vaksinasjon Dag 0	Dag 3 ± 1	Dag 7 ±	Dag 14 ±	Vaksinasjon Dag 21	Dag 24 ± 1	Dag 28 ± 1	Dag 35 ± 2	Dag 42 ± 4	6 måneder ± 0,5	12 måneder ± 0,5
**Dato**												
**Kontrollert egenmeldingsskjema for bivirkninger**												
**Tatt hematologiske prøver**												
**Tatt biokjemiske prøver**												
**Tatt immunologiske prøver**												
**Tatt spyttprøve**												
**Tatt blod til studien**												

*Deltager ID #*_________ *Kjønn*_________ *Alder*_________ *Initialer*___________

Vaksinasjonsdag 1. dose

INKLUSJONSKRITERIER: Alle setninger må besvares med JA for at personen skal kunne delta i studien

	**Ja**	Nei
Signert informert samtykke		
Frisk		
Kvinner: Bruker en pålitelig prevensjonsmetode så lenge studien pågår (fra 4 uker før 1. vaksinasjon til 4 uker etter 2. vaksinasjon)		
Kvinner: Negativ graviditetstest		
Screeningsprøver funnet OK av klinisk ansvarlig lege		

EKSKLUSJONSKRITERIER: Alle setninger må besvares med NEI for at personen skal kunne delta i studien

	**Nei**	**Ja**
Tidligere anafylaktisk sjokk eller alvorlige komplikasjoner etter vaksinering		
Kjent allergi overfor innholdstoffer i vaksine		
Feber >38°C i løpet av siste 72 timer		
Akutt luftveissykdom siste 7 dager		
Kvinner: gravid eller ammende		
Sykdom som kan påvirke evnen til å gjennomføre deltagelse i studien		
Fått blodprodukter eller immunglobulin parenteralt siste 3 måneder		
Kjent eller mistenkt immunsviktsykdom		
Bruk av medisiner som svekker immunforsvaret		
Bruk av immunstimulerende medikamenter		
Vaksinert i en 4 ukers periode før første vaksinasjon		
Vært med på andre kliniske utprøvninger siste måneden		

	**1. dose**
**Randomisering**	
**Vaksinasjonsdato**	
**Vaksinasjonsgruppe**	
**Vaksinasjonsdose og adjuvans (+/-)**	
**Vaksinasjonsside (hø/ve)**	
**Observasjon (45 minutter)** ***kryss av***	
**Reaksjon innen 45 minutter**	

Merkelapper fra vaksinasjonssprøyter

Deltager ID #__________ *Kjønn_________* *Alder_________* *Initialer___________*

Vaksinasjonsdag 2. dose

INKLUSJONSKRITERIER: Alle setninger må besvares med JA for at personen skal kunne delta i studien

	**Ja**	Nei
Frisk		
Kvinner: Bruker en pålitelig prevensjonsmetode så lenge studien pågår (fra 4 uker før 1. vaksinasjon til 4 uker etter 2. vaksinasjon)		
Kvinner: Negativ graviditetstest		
Screeningsprøver funnet OK av klinisk ansvarlig lege		

EKSKLUSJONSKRITERIER: Alle setninger må besvares med NEI for at personen skal kunne delta i studien

	**NEI**	JA
Tidligere anafylaktisk sjokk eller alvorlige komplikasjoner etter vaksinering		
Kjent allergi overfor innholdstoffer i vaksine		
Feber >38°C i løpet av siste 72 timer		
Akutt luftveissykdom siste 7 dager		
Kvinner: gravid eller ammende		
Sykdom som kan påvirke evnen til å gjennomføre deltagelse i studien		
Fått blodprodukter eller immunglobulin parenteralt siste 3 måneder		
Kjent eller mistenkt immunsviktsykdom		
Bruk av medisiner som svekker immunforsvaret		
Bruk av immunstimulerende medikamenter		
Vaksinert i en 4 ukers periode før første vaksinasjon		
Vært med på andre kliniske utprøvninger siste måneden		

	**2. dose**
**Kontrollert bivirkningsskjema**	
**Vaksinasjonsdato**	
**Vaksinasjonsgruppe**	
**Vaksinasjonsdose og adjuvans (+/-)**	
**Vaksinasjonsside (hø/ve)**	
**Observasjon (45 minutter)** ***kryss av***	
**Reaksjon innen 45 minutter**	

Merkelapper fra vaksinasjonssprøyter

*Deltager ID #*__________ *Kjønn*__________ *Alder*__________ *Initialer*__________

Resultat av blodprøver

		OK resultat?		kommentar
		Ja	Nei	
Dag –14- –12	Hematologiske prøver			
	Biokjemiske prøver			
	Immunologiske prøver			
Dag 3	Hematologiske prøver			
	Biokjemiske prøver			
	Immunologiske prøver			
Dag 21	Hematologiske prøver			
	Biokjemiske prøver			
	Immunologiske prøver			
Dag 24	Hematologiske prøver			
	Biokjemiske prøver			
	Immunologiske prøver			
Dag 42	Hematologiske prøver			
	Biokjemiske prøver			
	Immunologiske prøver			

*Deltager ID #*_________ *Kjønn*_________ *Alder*_________ *Initialer*___________

Bivirkninger vurdert av lege: medikament

DATO	ANTALL DAGER ETTER VAKSINASJON (1./2. dose)	BIVIRKNING	SAMMENHENG MED VAKSINASJON<br>1: relatert<br>2: ikke relatert	EVENTUELLE MEDIKAMENT TATT I FORBINDELSE MED BIVIRKNINGEN	VIDERE OPPFØLGING	KOMMENTARER<br>(Angi alvorlighetsgrad, og om hendelsen er meldt videre)	SIGN LEGE DATO

Norwegian Adverse Event Form

Deltager ID #:________ STARTDATO (Dag 0): ___/___/2006 Vaksinedose:_______

SKJEMA FOR EGENRAPPORTERING AV BIVIRKNINGER/REAKSJONER ETTER INFLUENSAVAKSINASJON MED H5N1 VAKSINE

MERK AV I DE RUBRIKKENE SOM PASSER. Gradér fra 1-3
1= Svake/ ubetydelige symptomer som ikke gir særlige plager.
2= Symptomer som er så besværlige at de påvirker den daglige aktiviteten, men som ikke krever legetilsyn.
3= Sterke eller vedvarende symptomer som i høy grad påvirker den daglige aktiviteten, eller som krever legetilsyn.
Tom rubrikk betyr "intet å melde". DET ER PLASS TIL EVT. KOMMENTARER PÅ BAKSIDEN AV SKJEMAET. Reaksjoner som ikke er nevnt i tabellen: Bruk kommentarfeltet. Angi også i samme kommentarfelt om du har tatt noen medisiner som en følge av reaksjonene, og i så fall hvilke medisiner.
Ved akutt legebehov, se telefonliste på baksiden av skjemaet. Ved bruk av annen lege: Meld fra om at du er med i denne studien.
Skjemaet bringes med til klinikken og vises legen ved hvert frammøte (dagene skravert på skjemaet).

	Lokale symptomer på injeksjonsted					Generelle symptomer										
Dag etter vaksinasjon	Rødhet	Hevelse	Smerte	Liten blodutredelse	Hard hud	Feber >38°C (angi temp)	Uvel (beskriv under)	Skjelving	Tretthet	Hode-pine	Svetting	Muskel-verk	Ledd-smerter	Luftveis-symptomer (beskriv under)	Diaré	Andre hendelser
0																
1																
2																
3																
4																
5																
6																
7																
8																
9																
10																
11																
12																
13																
14																
15																
16																
17																
18																
19																
20																
21																

Telefon for medisinsk assistanse i kontortiden: Dr. Haak[illegible]jursen 55 97 29 24

Telefon for medisinsk assistanse utenfor kontortid: Dr. H[illegible]kon Sjursen 55 13 54 80 (mobil 901 46 407[illegible] [illegible]ller sentralbord Haukeland Universitetssjukehus 55 97 50 00 [illegible]pør etter vakthavende lege på infeksjonsmed. [illegible]vd.

Eventuelle kommentarer:

Dato	Dag etter vaksinasjon	Kommentar
